# A novel protein purification scheme based on salt inducible self-assembling peptides

**DOI:** 10.1186/s12934-023-02229-5

**Published:** 2023-10-30

**Authors:** Guang Zeng, Yinzhen Zheng, Ya Xiang, Run Liu, Xiaofeng Yang, Zhanglin Lin

**Affiliations:** 1 School of Biology and Biological Engineering, South China University of Technology, 382 East Outer Loop Road, University Park, Guangzhou, 510006 China; 2 Shenzhen Institute of Synthetic Biology, Shenzhen Institutes of Advanced Technology, Chinese Academy of Sciences, Shenzhen, 518055 China

**Keywords:** Protein expression and purification, Controllable aggregating tag, Salt-inducible self-assembling peptide, Intein, *Escherichia coli*

## Abstract

**Background:**

Protein purification remains a critical need for biosciences and biotechnology. It frequently requires multiple rounds of chromatographic steps that are expensive and time-consuming. Our lab previously reported a cleavable self-aggregating tag (cSAT) scheme for streamlined protein expression and purification. The tag consists of a self-assembling peptide (SAP) and a controllable self-cleaving intein. The SAP drives the target protein into an active aggregate, then by intein-mediated cleavage, the target protein is released. Here we report a novel cSAT scheme in which the self-assembling peptide is replaced with a salt inducible self-assembling peptide. This allows a target protein to be expressed first in the soluble form, and the addition of salt then drives the target protein into the aggregated form, followed by cleavage and release.

**Results:**

In this study, we used MpA (MKQLEDKIEELLSKAAMKQLEDKIEELLSK) as a second class of self-assembling peptide in the cSAT scheme. This scheme utilizes low salt concentration to keep the fusion protein soluble, while eliminating insoluble cellular matters by centrifugation. Salt then triggers MpA-mediated self-aggregation of the fusion, removing soluble background host cell proteins. Finally, intein-mediated cleavage releases the target protein into solution. As a proof-of-concept, we successfully purified four proteins and peptides (human growth hormone, 22.1 kDa; LCB3, 7.7 kDa; SpyCatcherΔN-ELP-SpyCatcherΔN, 26.2 kDa; and xylanase, 45.3 kDa) with yields ranging from 12 to 87 mg/L. This was comparable to the classical His-tag method both in yield and purity (72–97%), but without the His-tag. By using a further two-step column purification process that included ion-exchange chromatography and size-exclusion chromatography, the purity was increased to over 99%.

**Conclusion:**

Our results demonstrate that a salt-inducible self-assembling peptide can serve as a controllable aggregating tag, which might be advantageous in applications where soluble expression of the target protein is preferred. This work also demonstrates the potential and advantages of utilizing salt inducible self-assembling peptides for protein separation.

**Supplementary Information:**

The online version contains supplementary material available at 10.1186/s12934-023-02229-5.

## Background

Protein purification is a fundamental technology for both research and commercial applications of proteins [1–4]. Generally, to purify a target protein from the background host cell proteins (HCPs), multiple rounds of chromatographic steps are applied based on the properties of proteins, such as isoelectric point (pI), hydrophobicity and size [5, 6]. An alternative approach involves attaching an affinity tag, e.g., His-tag [7], GST-tag [8] and FLAG-tag [9], to the protein of interest. This allows the protein to be specifically isolated using affinity chromatography [10]. However, the resins used in this process are often expensive and have low capacities (Additional file 2: Table S1) [1, 11]. Additionally, for therapeutic proteins, the tags must be removed using endopeptidases. This increases production costs and time, particularly on a larger scale [3, 11].

Over the past two decades, self-assembling peptide (SAP) such as EAK16 and RADA16 have been found to possess a remarkable ability to spontaneously form well-ordered nanofibers and stable membranes [12, 13]. These studies have revolutionized our understanding of peptides as a distinct class of materials that can be precisely designed and synthesized with excellent physical and structural properties [14]. The immense versatility of self-assembling peptides provides vast potential for various biotechnology and biomaterials applications [15], including cell culture [16], drug delivery and controlled release [17, 18], antibacterial and anticancer materials [19, 20], and biomimetic mineralization [21, 22]. For example, RADA16 is now sold marketed as a hemostatic agent for surgery (PuraStat®) [23].

Our lab has previously developed a cleavable self-aggregating tag (cSAT) scheme for protein expression and purification [24–27]. This tag consists of a SAP of 8–30 amino acids and a controllable self-cleaving intein. In this scheme, the SAP drives the target protein into an active aggregate, enabling fast removal of most of the HCPs through centrifugation. Then, by intein-mediated cleavage, the target protein is released, with yields ranging from 3 to 89 mg/L and purity ranging from 46 to 90% [27], which are comparable to those of the classical His-tag method [28]. We have further demonstrated that due to the removal of most of the HCPs, it is possible to achieve high purity (> 99%) for the target protein using a standard two-step column purification comprising ion-exchange chromatography (IEC) and size-exclusion chromatography (SEC) [26, 27]. For instance, at the shake flask scale, the yield of free human growth hormone (hGH) was 73 mg/L [27], which is higher than the yield of His-tagged hGH purified by immobilized metal affinity chromatography, IEC, and SEC (40 mg/L) [29]. Notably, the yield of purified free hGH using a 30-liter fermenter was estimated to be 2.0 g/L [27].

Recently, we noted that a short peptide CpA (CKQLEDKIEELLSKAACKQLEDKIEELLSK, 30 aa) remained disordered and soluble at a low salt concentration (10 mM Tris-HCl, pH 8.0), but formed a helical fiber under 3 M NaCl [30]. It inspired us to explore the use of CpA and two other closely related variants, IpA (IKQLEDKIEELLSKAAIKQLEDKIEELLSK) [30] and MpA (MKQLEDKIEELLSKAAMKQLEDKIEELLSK), in our cSAT scheme. Using hGH [31] as the model protein, we indeed found that the fusion protein (SAP-intein-hGH) was soluble in a low salt cell lysis buffer (20 mM Tris-HCl, 1 mM EDTA, pH 8.0), but the fusion could be selectively precipitated and separated from HCPs by adding salt (0.7 M Na_2_SO_4_, or 3 M NaCl, or 0.7 M (NH_4_)_2_SO_4_). The hGH was then released into the solution by intein-mediated cleavage as previously described [27]. Compared to the original cSAT scheme, this salt inducible cSAT (icSAT) scheme has two advantages: (1) it allows for the isolation of the target protein from the supernatant of the cell lysate, thereby reducing the contamination from membrane proteins and phospholipids in the insoluble cell matters, and (2) it is particularly beneficial in applications where the target protein is preferred to be expressed as a soluble form.

We investigated the usefulness of the icSAT scheme by selecting a series of proteins and peptides, including a therapeutic protein, hGH (22.1 kDa) [31], a peptide-based severe acute respiratory syndrome coronavirus 2 (SARS-CoV-2) inhibitor, LCB3 (7.7 kDa) [32], a multivalent scaffold protein, SpyCatcherΔN-ELP-SpyCatcherΔN (26.2 kDa) [33–35], and an industrial enzyme, xylanase (45.3 kDa) [36]. Furthermore, we assessed whether this icSAT scheme could be integrated with a standard two-step column purification (i.e., IEC and SEC) to attain high purity (> 99%). Our findings suggest that the icSAT scheme is an economical, reliable, and standardizable method for the production of proteins or peptides in *Escherichia coli* (*E. coli*). Furthermore, this study demonstrates the potential of inducible self-assembling peptides for greatly streamlining the protein purification process.

## Results

### Establishment of the icSAT purification method

**Fig. 1:**
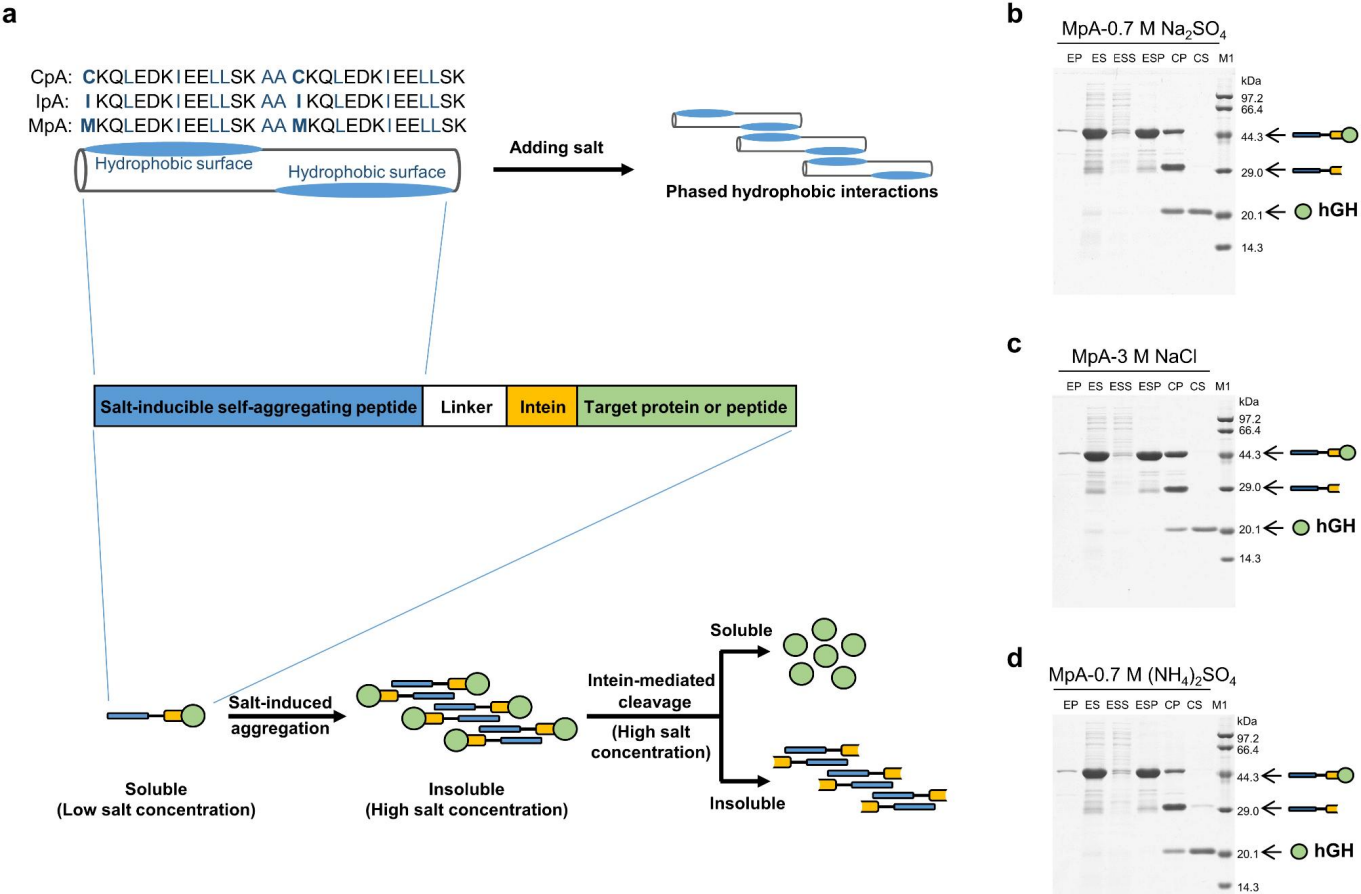
The icSAT scheme for the purification of target proteins. **a** Schematic diagram of the icSAT scheme. The sequences of three salt-inducible self-assembling peptides (CpA, IpA and MpA) are shown in Fig. 1a, with hydrophobic residues highlighted in blue for clarity. The proposed icSAT tag is composed of one of the three peptides (CpA, IpA or MpA), a linker (i.e., PT linker [24]), and an intein (i.e., *Mtu* ΔI-CM). When the salt concentration is increased to a suitable level, the icSAT tag induces the fusion protein to form an insoluble aggregate that can be conveniently harvested by centrifugation. Subsequent treatment of the harvested aggregate with intein-mediated cleavage releases the target protein into solution. **b-d** Purification of hGH from the fusion protein MpA-*Mtu* ΔI-CM-hGH with three high-salt conditions. EP: precipitate of the cell lysate after expression, ES: supernatant of the cell lysate after expression, ESS: supernatant of salt-induced aggregation, ESP: precipitate of salt-induced aggregation, CP: precipitate of intein-mediated cleavage, CS: supernatant of intein-mediated cleavage, M1: protein marker. (**b**) 0.7 M Na_2_SO_4_, (**c**) 3 M NaCl, (**d**) 0.7 M (NH_4_)_2_SO_4_

As previously reported [30], the peptide CpA (CKQLEDKIEELLSKAACKQLEDKIEELLSK) was designed from the leucine zipper motif GCN4 (MKQLEDKVEELLSK). The insertion of two alanine residues (amino acids underlined) causes a phase shift in the C-terminal hydrophobic surface in relation to the N-terminal hydrophobic surface. Addition of salt enhances the hydrophobic interaction between peptides, leading to self-assembly of free peptides and the formation of helical fibers. IpA (IKQLEDKIEELLSKAAIKQLEDKIEELLSK) is a variant of CpA that displays increased salt-responsiveness, due to the presence of isoleucine in place of cysteine at the first positions of two of its heptad repeats (amino acid replacements underlined) [30]. Therefore, we designed a second variant, MpA (MKQLEDKIEELLSKAAMKQLEDKIEELLSK), in which the two cysteines were mutated to the nonpolar amino acid, methionines, to optimize for microbial expression and explore enhanced salt-responsiveness. Utilizing this salt-inducible self-assembling feature, we designed the icSAT tag, which consists of the salt-inducible self-assembling peptide CpA/IpA/MpA along with the intein *Mtu* ΔI-CM [37], as illustrated in Fig. 1a.

We first used hGH [31] as the target protein to construct three fusion proteins CpA-*Mtu* ΔI-CM-hGH, IpA-*Mtu* ΔI-CM-hGH and MpA-*Mtu* ΔI-CM-hGH. After cell lysis in a low salt buffer B1 (20 mM Tris-HCl, 1 mM EDTA, pH 8.0), the soluble fusion proteins were isolated from the insoluble cell matters by centrifugation. Then the salt was added to a final concentration of 0.7 M Na_2_SO_4_, or 3 M NaCl, or 0.7 M (NH_4_)_2_SO_4_, resulting in 91 ± 6% of the soluble fusion proteins self-associating into insoluble aggregates (Fig. 1b-d and Additional file 1: Fig. S1). These aggregates were then separated from the soluble HCPs by a second centrifugation step, and re-suspended in a high salt cleavage buffer B4 (20 mM Bis-Tris, 10 mM Na_2_HPO_4_, 1.8 mM KH_2_PO_4_, 0.7 M Na_2_SO_4_/3 M NaCl/0.7 M (NH_4_)_2_SO_4_, 2.7 mM KCl, 2 mM EDTA, pH 6.2) to initiate the intein-mediated cleavage reaction and release hGH. With no additional purification, the average yield for three salt conditions was 83 ± 26 mg/L with a purity of 84 ± 14% (Table 1), which was comparable with those obtained using the cSAT scheme (89 mg/L with a purity of 90% also in the form of free hGH) [27] or the His-tag method (84 mg/L with a purity of 70% but in the form of His-tagged hGH) [29].

**Table 1:** Application of the icSAT scheme for the purification of different proteins and peptides

Type	Target protein	Intein	0.7 M Na_2_SO_4_		3 M NaCl		0.7 M (NH_4_)_2_SO_4_		Specific activity of purified proteins and peptides	Specific activity of commercial or literature-sourced proteins and peptides
			Yield^a^ (mg/L)	Purity^b^ (%)	Yield^a^ (mg/L)	Purity^b^ (%)	Yield^a^ (mg/L)	Purity^b^ (%)		
Therapeutic protein	hGH	*Mtu* ΔI-CM	87 ± 6	97 ± 1	81 ± 12	94 ± 7	127 ± 16	88 ± 7	0.13–0.19 nM^c^	0.15 nM^d^
Peptide	LCB3	*Mtu* ΔI-CM	12 ± 3	72 ± 1	10 ± 6	71 ± 16	4 ± 3	33 ± 8	0.31–0.43 nM^c^	< 1 nM [32]
Multivalent scaffold protein	SpyCatcherΔN-ELP-SpyCatcherΔN	*Mtu* ΔI-CM	40 ± 9	96 ± 1	45 ± 8	98 ± 1	40 ± 6	85 ± 4	~ 99%^e^	~ 80%^f^ [34]
Industrial enzyme	xylanase	*Mtu* ΔI-CM	32 ± 4	79 ± 1	24 ± 4	67 ± 3	18 ± 1	59 ± 4	12.37–26.19 units/mg	≥ 2.50 units/mg^g^
		*Mxe* GyrA	84 ± 6	87 ± 6	35 ± 13	49 ± 15	37 ± 6	54 ± 7	13.31–28.60 units/mg	

^a^Yield of target proteins or peptides after intein-mediated cleavage per liter of culture. ^b^Purity is calculated as the mass ratio of target proteins or peptides to total proteins in the supernatant after intein-mediated cleavage, estimated using densitometry analysis software ImageJ. ^c^The dissociation constants of hGH for binding to the hGH receptor and of LCB3 for binding to the SARS-CoV-2 spike receptor were measured using the respective commercial receptors. Prior to the binding assays, the three icSAT-purified hGH proteins and LCB3 peptides were subject to ion-exchange chromatography to achieve further purification. ^d^The dissociation constant of commercial hGH for binding to the hGH receptor in this study. ^e^The covalent reconstitution between SpyCatcherΔN-ELP-SpyCatcherΔN and LCB3-SpyTag was measured using a 3-fold excess of LCB3-SpyTag, following the protocol recommended by Paul J Wichgers Schreur [35]. ^f^The covalent reconstitution was that between SpyCatcher and SpyTag-MBP. ^g^The activity of xylanase available commercially from Sigma-Aldrich

Additional file 2: Table S2 shows that MpA had the highest yield of soluble fusion protein (611 ± 32 mg/L) and the highest yield and purity for free hGH. Among the three high-salt conditions, 0.7 M Na_2_SO_4_-mediated purification performed the best in terms of the purity (97 ± 1%) with a considerable yield of hGH (87 ± 6 mg/L), while 3 M NaCl-mediated purification resulted in a similar yield (81 ± 12 mg/L) and purity (94 ± 7%), and 0.7 M (NH_4_)_2_SO_4_-mediated purification obtained the highest yield of hGH (127 ± 16 mg/L) with a slightly lower purity (88 ± 7%). Therefore, we chose MpA and the three high-salt conditions for further study, with 0.7 M Na_2_SO_4_ being the preferred high-salt condition.

To confirm that the three high-salt conditions utilized in the MpA-mediated icSAT scheme did not impair the affinity of hGH for binding to the hGH receptor, biolayer interferometry (BLI) measurements were performed. As present in Table 1 and Additional file 1: Fig. S2, the dissociation constants (*K*_D_) of all three purified hGH were in the range of 0.13–0.19 nM, which is comparable to those of commercial hGH (0.15 nM), indicating that the three high-salt conditions tested did not have noticeable adverse effects on the activity of hGH.

During the expression and purification of hGH using this icSAT scheme, we observed that the fusion protein MpA-*Mtu* ΔI-CM-hGH was cloudy when using the regular lysis buffer with 500 mM NaCl (20 mM Tris-HCl, 500 mM NaCl, 1 mM EDTA, pH 8.5) [24, 27, 38], but when we used a low salt buffer B1 (20 mM Tris-HCl, 1 mM EDTA, pH 8.0, with a ionic strength of ~ 0.011 M), a soluble form of MpA-*Mtu* ΔI-CM-hGH fusion protein was obtained. To probe the state in which the MpA-tagged hGH was expressed inside *E. coli*, we prepared buffer B8 (20 mM Tris-HCl, 225 mM NaCl, 1 mM EDTA, pH 8.0, with a similar ionic strength to that of the intracellular ionic strength of *E. coli*, ~ 0.237 M) for cell lysis [39]. Following cell lysis in this buffer, 68% of MpA-*Mtu* ΔI-CM-hGH fusion was found in the precipitate of the cell lysate (Additional file 1: Fig. S3). However, the precipitate was entirely redissolved in the low salt buffer B1 by sonication (95%, Additional file 1: Fig. S3).

The distribution of the MpA-tagged fusion protein in vivo was also analyzed by fluorescence confocal microscopy with mCherry as the model protein. As shown in Additional file 1: Fig. S4a, when mCherry was expressed alone, cells exhibited uniform red fluorescence throughout the cytoplasm. For the MpA-tagged mCherry, a partially localized distribution of fluorescence was observed in the cells (Additional file 1: Fig. S4b). This confirmed the in vivo formation of both the active aggregate and soluble forms of MpA-mCherry. We lysed the MpA-tagged mCherry cells in buffer B8 and found that 30% of MpA-tagged mCherry fusion was insoluble (Additional file 1: Fig. S5), and this insoluble portion of fusion could also be redissolved in buffer B1 by sonication (95%, Additional file 1: Fig. S5).

In summary, we have established a straightforward protein purification method that effectively isolates hGH from the supernatant of *E. coli* cell lysate. Our method resulted in high yields, purity, and maintained the activity of the protein.

### Extension of the icSAT method to other proteins and peptides

**Fig. 2:**
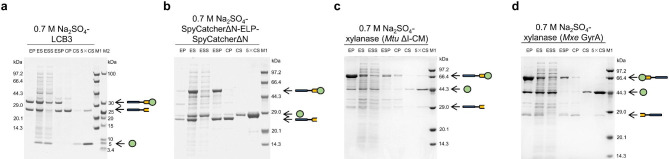
Purification of LCB3/SpyCatcherΔN-ELP-SpyCatcherΔN/xylanase with 0.7 M Na_2_SO_4_. M1 and M2: protein marker. EP: precipitate of the cell lysate after expression, ES: supernatant of the cell lysate after expression, ESS, supernatant of salt-induced aggregation, ESP: precipitate of salt-induced aggregation, CP: precipitate of intein-mediated cleavage, CS and 5×CS: supernatant of intein-mediated cleavage, the 5×CS was loaded at 5 times the amount of the CS. **a** LCB3. **b** SpyCatcherΔN-ELP-SpyCatcherΔN. **c-d** Purification results for xylanase from the two fusion proteins, MpA-*Mtu* ΔI-CM-xylanase (**c**) and xylanase-*Mxe* GyrA-MpA (**d**), individually

To further evaluate the efficacy of the icSAT scheme, we extended its application to other proteins and peptides, including LCB3 [32], a peptide-based SARS-CoV-2 inhibitor, SpyCatcherΔN-ELP-SpyCatcherΔN [33–35], a multivalent scaffold protein, and xylanase [36], an industrial enzyme. The resulting fusion proteins (MpA-*Mtu* ΔI-CM-LCB3/SpyCatcherΔN-ELP-SpyCatcherΔN/xylanase) were successfully produced in the supernatants of cell lysates. As shown in Table 1 and Fig. 2a-c, the yields and purities of the target proteins in their free forms varied but were within a same scale compared to the His-tagged counterparts obtained using the His-tag method. For example, using 0.7 M Na_2_SO_4_ as the high-salt condition, the yield of free LCB3 was 12 ± 3 mg/L with a purity of 72 ± 1%, while the yield of His-tagged LCB3 was 68 mg/L with a purity of 88% (data not shown). Similarly, the yield of free xylanase was 32 ± 4 mg/L with a purity of 79 ± 1%, while the yield of His-tagged xylanase was 8 mg/L with a purity of 98% [40]. It is important to note that the former were free proteins with authentic termini while the latter were all His-tagged proteins. Removing of the His-tag would significantly reduce the yields of the free proteins in the His-tag method. In addition to 0.7 M Na_2_SO_4_, we purified the three proteins and peptides with 3 M NaCl and 0.7 M (NH_4_)_2_SO_4_, and evaluated them for yields (4–45 mg/L) and purities (33–98%) (Table 1 and Additional file 1: Fig. S6a-c and Fig. S7a-c). All proteins or peptides purified using three high-salt conditions were assayed. The *K*_D_ of LCB3 were in the range of 0.31–0.43 nM (Table 1, Additional file 1: Fig. S8), which is comparable to the literature reported (< 1 nM) [32]. The covalent reconstitutions of SpyCatcherΔN-ELP-SpyCatcherΔN were approximately 99% (Table 1, Additional file 1: Fig. S9), which is comparable to the literature reported [33, 34, 41]. The enzyme activities of xylanase were in the range of 12.37–26.19 units/mg (Table 1, Additional file 1: Fig. S10), which is higher than those of the commercial xylanase (≥ 2.50 units/mg) from Sigma-Aldrich.

Additionally, to test whether the widely used intein *Mxe* GyrA [42] is suitable for the icSAT scheme, we chose xylanase as the target protein. This form of xylanase had three additional amino acid residues (MRM) at its C-terminus, and an additional methionine residue encoded as the start codon at its N-terminus [26]. Such modifications are generally acceptable for an industrial enzyme. In the case of xylanase, replacing the C-terminal cleavage intein *Mtu* ΔI-CM with the N-terminal cleavage intein *Mxe* GyrA increased the yield to 84 ± 22 mg/L with a purity of 87 ± 6% (Table 1; Fig. 2d).

Taken together, these results demonstrated that the MpA-mediated icSAT method allowed reliable purification of a diverse range of proteins and peptides with high activities.

A set of other controllable aggregating proteins has been repoted in the literature [43], which includes the heat/salt-inducible ELPs (elastin-like polypeptides, ~ 40 kDa) [44], and calcium-inducible RTX (repeat-in-toxin domain, 14 kDa) [45] and Annexin B1 (an annexin protein from *Cysticercus cellulosae*, 36 kDa) [46]. While there is extensive literature available on ELPs, there is limited published work on the use of RTX and Annexin B1 as tags for protein expression. We tested their potential as tags for hGH production. As shown in Additional file 1: Fig. S11 and Additional file 2: Table S3, The N-terminal RTX tag had a negative impact on cell growth, resulting in a yield of only 6 ± 1 mg/L of hGH with a purity of 84 ± 3%. On the other hand, the Annexin B1 tag led to a yield of 8 ± 3 mg/L of hGH and a purity of only 18 ± 6%. This low purity was due to approximately two-thirds of the Annexin B1-*Mtu* ΔI-CM fragment failing to self-associate into a precipitate after the addition of 20 mM calcium chloride.

### Verification of standard purification protocol for the target protein or peptide

**Fig. 3:**
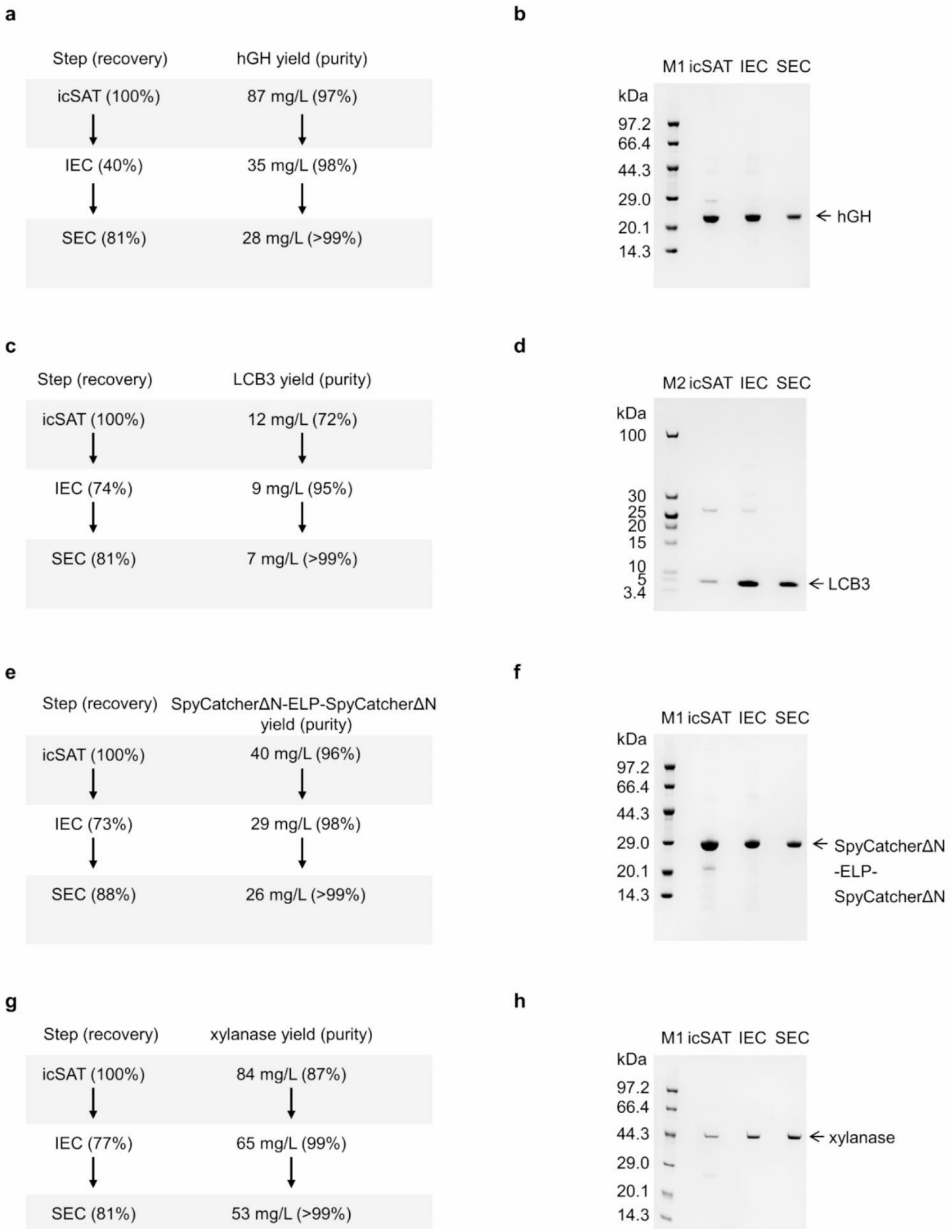
Two-step column purification for the four targets, hGH, LCB3, SpyCatcherΔN-ELP-SpyCatcherΔN and xylanase. (**a, c, e, g**) Process summary and (**b, d, f, h**) SDS-PAGE results for the four targets individually. M1 and M2: protein marker

Similar to the original cSAT scheme [27], we found the residual MpA-*Mtu* ΔI-CM fragment as the major impurity in the icSAT scheme. Therefore, we adopted the two-step chromatography (IEC and SEC) used in the cSAT scheme [27] to further purify the four aforementioned proteins and peptides. The resins for IEC and SEC were chosen based on the pI and the molecular weights (MW) of the target proteins and peptides, respectively (Additional file 2: Table S4). Specifically, in the IEC step, the Capto Q resin with buffer B9 (20 mM Tris-HCl, pH 7.2) was used for hGH, LCB3, and SpyCatcherΔN-ELP-SpyCatcherΔN, with their pI ranging from 4.9 to 5.3. For xylanase, whose pI is 8.2, Capto S resin with buffer B10 (50 mM sodium phosphate, pH 7.0) was used. In the SEC step, the Superdex 75 resin (with fractionation range of 3–70 kDa) was used for all four targets whose MW ranged from 7.7 to 45.9 kDa. Target proteins were eluted following the manufacturers’ recommended protocols (for chromatograms, see Additional file 1: Fig. S12). As shown in Fig. 3, we obtained yields of 28 mg/L, 7 mg/L, 26 mg/L and 53 mg/L for hGH, LCB3, SpyCatcherΔN-ELP-SpyCatcherΔN and xylanase, respectively. The results of the protein gel (Fig. 3) and the RP-HPLC assay (Additional file 1: Fig. S13) indicated that the purities of the four target proteins or peptides were above 99%. However, the recovery rates for IEC were lower, ranging from 40 to 77%, compared to those of the cSAT scheme (90–91%) [27]. In contrast, the recovery rates for SEC ranged from 81 to 88%, which were comparable to those of the cSAT scheme (79–82%) [27]. Overall, the two-step chromatography introduced in this study was able to effectively purify all four target proteins and peptides with a purity greater than 99%.

## Discussion

In this work, we utilized the salt-inducible self-assembling peptide MpA to develop a novel approach for protein expression and purification in *E. coli.* This approach dramatically simplifies the isolation of target proteins from the background host cell proteins and other biomolecules without the need for affinity resins or subsequent tag removal. Compared with these reported controllable aggregating protein tags, such as ELP (~ 40 kDa), RTX (14 kDa) and Annexin B1 (36 kDa), the peptide MpA (3.5 kDa) used in this work is much shorter in size, with high-yield bacterial expression of MpA-tagged fusion proteins (i.e., in the range of 135–611 mg/L), and much simpler aggregating strategies. For example, in contrast, the ELPs purification requires inverse transition cycling of cooling and heating steps [44, 47]. The facile and effective removal of HCPs in this MpA-mediated approach enables a standard two-step column purification, including IEC and SEC, for achieving a high purity level of > 99% for target proteins.

It has been estimated that the cost of purifying ELPs is approximately 10% of the classical His-tag method [28]. As our icSAT scheme is even simpler, it should provide comparable cost savings at a minimum. However, when we combined the icSAT scheme with a two-step standard column purification (i.e., IEC and SEC), we noticed a decrease in the efficiency of IEC compared to the previous cSAT scheme [27]. Specifically, the yield for hGH recovery in the IEC step declined to 40% for icSAT, versus 91% for cSAT [27]. This difference may be attributed to the varying pI values between hGH (pI 5.3) and the main residual MpA-*Mtu* ΔI-CM fragment (pI 5.7) in icSAT, as well as the L_6_KD-*Mtu* ΔI-CM fragment (pI 6.0) in cSAT [27]. Therefore, one future task for improving icSAT is to revise the fusion design to change the pI of the residual SAP-intein fragment.

In summary, this protein purification method demonstrates the potential and advantages of utilizing salt inducible self-assembling peptides for protein separation, and it should reduce both production cost and process development time.

## Methods

### Strains and materials

The strains *E. coli* DH5α and *E. coli* BL21 (DE3), and pET30a plasmid were obtained from Novagen (Madison, WI, USA). DNA sequences encoding LCB3 [32], SpyCatcherΔN-ELP-SpyCatcherΔN [33–35], and xylanase [36] were optimized for expression in *E. coli* and synthesized by Sangon (Shanghai, China), while those encoding RTX and Annexin B1 were synthesized by Ruibiotech (Guangzhou, China). Oligonucleotides were synthesized by Sangon (Guangzhou, China) and listed in Additional file 2: Table S5. The DNA sequencing was performed by Sangon (Guangzhou, China) or Tsingke (Guangzhou, China). Restriction enzymes and DNA polymerases were purchased from New England Biolabs (Beverly, MA, USA). The plasmid mini-preparation kit was obtained from Tiangen (Beijing, China), while the HiPure gel pure DNA micro kit was from Magen Biotechnology (Guangzhou, China). Commercial hGH (Jintropin®, GeneScience Pharmaceuticals, China) was purchased from the Third Affiliated Hospital, Sun Yat-sen University (Guangzhou, China). Dithiothreitol (DTT), xylan, and xylose were purchased from Sigma-Aldrich (Shanghai, China). For the detection of protein or peptide binding, Amine Reactive 2nd Generation (AR2G) biosensors were obtained from ForteBio (Menlo Park, CA). Recombinant human Growth hormone receptor protein was purchased from Abcam (Cambridge, UK), while the SARS-CoV-2 spike protein (RBD, His & Avi tag) was purchased from GenScript (Nanjing, China).

### Plasmid construction

Oligonucleotide design for PCR-based gene synthesis was performed using DNAWorks 3.2 (https://hpcwebapps.cit.nih.gov/dnaworks/) [48], and the MpA-PT linker and GS linker-MpA DNA fragments were synthesized via PCR-based gene synthesis. The nucleotide sequence and description for primers used in this study were shown in Additional file 2: Table S5. The *Mtu* ΔI-CM-hGH DNA fragment was amplified from the plasmid pET32a-L_6_KD-*Mtu* ΔI-CM-hGH [27] and then assembled with the MpA-PT linker DNA fragment by overlap extension PCR. The resulting product was inserted into the *Nde*I and *Xho*I sites of pET30a, generating the plasmid pET30a-MpA-*Mtu* ΔI-CM-hGH.

To construct the plasmids pET30a-CpA-*Mtu* ΔI-CM-hGH and pET30a-IpA-*Mtu* ΔI-CM-hGH, desired amino acid substitutions were introduced using primer-mediated site-directed mutagenesis. More specifically, the plasmid pET30a-CpA-*Mtu* ΔI-CM-hGH was constructed by utilizing Gibson assembly [49], wherein the CpA-*Mtu* ΔI-CM-hGH-*KanR* and *lacI*-CpA DNA fragments that were amplified from pET30a-MpA-*Mtu* ΔI-CM-hGH were utilized. Likewise, the plasmid pET30a-IpA-*Mtu* ΔI-CM-hGH was also generated via Gibson assembly [49], using the IpA-*Mtu* ΔI-CM-hGH-*KanR* and *lacI*-IpA DNA fragments that were amplified from pET30a-MpA-*Mtu* ΔI-CM-hGH.

To construct the plasmids pET30a-MpA-*Mtu* ΔI-CM-SpyCatcherΔN-ELP-SpyCatcherΔN, pET30a-MpA-*Mtu* ΔI-CM-LCB3 and pET30a-MpA-*Mtu* ΔI-CM-xylanase, the target protein and peptide genes were individually amplified by PCR and then inserted downstream from the *Mtu* ΔI-CM using Gibson assembly [49]. To construct the plasmid pET30a-xylanase-*Mxe* GyrA-MpA, the *Mxe* GyrA DNA fragment was amplified from the plasmid pET30-hGH-*Mxe* GyrA-L_6_KD [26]. This fragment was then assembled with the GS linker-MpA DNA fragment by overlap extension PCR. The resulting product, along with the *KanR-lacI* DNA fragment amplified from pET30a and the xylanase gene, was assembled by Gibson assembly [49] to obtain the plasmid of interest.

The plasmid pET30a-MpA-mCherry was constructed using Gibson assembly [49] by individually amplifying the *lacI*-MpA DNA fragment and the mCherry-*KanR* DNA fragment from the plasmids pET30a-MpA-*Mtu* ΔI-CM-hGH and pET30a-Spy-RFP [41], respectively.

To construct the plasmids pET30a-RTX-*Mtu* ΔI-CM-hGH and pET30a-Annexin B1-*Mtu* ΔI-CM-hGH, the DNA fragments encoding RTX [45] and Annexin B1 [46] were amplified by PCR individually and then inserted upstream of the *Mtu* ΔI-CM-hGH using Gibson assembly [49].

### Protein expression

The protein expression methods used in this work were modified from previous reports [27, 45, 46]. Briefly, *E. coli* BL21 (DE3) cells containing the target plasmids for expressing CpA/IpA/MpA/Annexin B1-*Mtu* ΔI-CM-hGH, MpA-*Mtu* ΔI-CM-LCB3/SpyCatcherΔN-ELP-SpyCatcherΔN, MpA-mCherry, and mCherry were inoculated into lysogeny broth (LB) medium supplemented with 50 mg/L kanamycin. For the expression of MpA-*Mtu* ΔI-CM-xylanase, xylanase-*Mxe* GyrA-MpA, and RTX-*Mtu* ΔI-CM-hGH, *E. coli* BL21 (DE3) cells containing the target plasmids were inoculated into Terrific Broth (TB) medium supplemented with 50 mg/L kanamycin. The *E. coli* cultures were incubated at 37 °C with shaking. When the OD_600_ reached 0.4–0.6, the incubation temperature was reduced to 18 °C. After 15–30 min, isopropyl β-D-1-thiogalactopyranoside (IPTG) was added to a final concentration of 0.2 mM to initiate protein expression. The cultures were incubated for an additional 24 h at 18 ℃ before the cells were harvested by centrifugation at 4,000 *g* for 20 min at 4 °C.

### Protein purification

For the *Mtu* ΔI-CM-mediated cleavage of icSAT scheme, the harvested cell pellets were re-suspended in buffer B1 (20 mM Tris-HCl, 1 mM EDTA, pH 8.0) and sonicated on ice using an Ultrasonic crasher (Scientz JY92-IIN, Ningbo, China). The soluble fractions were isolated from the cell lysate through centrifugation at 15,000 *g* for 20 min at 4 °C. The soluble fractions were either mixed with an equal volume of buffer B2 (20 mM Tris-HCl, 1.4 M Na_2_SO_4_ or 1.4 M (NH_4_)_2_SO_4_, 1 mM EDTA, pH 8.0) solution or with NaCl powder to a final concentration of 3 M. The resulting mixture was incubated at 4 °C for 1 h and subsequently centrifuged at the same temperature for 20 min. The supernatant was carefully removed, and the precipitate was re-suspended in buffer B3 (20 mM Tris-HCl, 0.7 M Na_2_SO_4_/3 M NaCl/0.7 M (NH_4_)_2_SO_4_, 1 mM EDTA, pH 8.0), reserving an aliquot for analysis by SDS-PAGE. The sample was then centrifuged again to pellet the insoluble fusion protein, and the pellet was re-suspended in buffer B4 (20 mM Bis-Tris, 10 mM Na_2_HPO_4_, 1.8 mM KH_2_PO_4_, 0.7 M Na_2_SO_4_/3 M NaCl/0.7 M (NH_4_)_2_SO_4_, 2.7 mM KCl, 2 mM EDTA, pH 6.2) and incubated at 25 °C for 24 h to allow the intein-mediated cleavage reaction. Finally, the soluble fraction was collected by centrifugation.

For the *Mxe* GyrA-mediated cleavage of icSAT scheme (used only for xylanase), the sample containing the salt-induced aggregate was first re-suspended and reserved in an aliquot. Then it was centrifuged again to pellet the insoluble fusion protein, which was subsequently re-suspended in buffer B5 (20 mM Tris-HCl, 0.7 M Na_2_SO_4_/3 M NaCl/0.7 M (NH_4_)_2_SO_4_, 1 mM EDTA, 40 mM DTT, pH 8.0) and incubated at 25 °C for 24 h to allow the intein-mediated cleavage reaction. Finally, the soluble fraction was collected by centrifugation.

For the RTX-mediated protein purification, the protocol was modified from previous reports [45]. Harvested cell pellets were first re-suspended in buffer B12 (50 mM Tris-HCl, pH 7.4) and subsequently sonicated on ice. The soluble fractions were isolated from the cell lysate by centrifugation at 15,000 *g* for 20 min at 4 °C. The precipitation of the RTX fusion proteins was initiated by adding 2 M CaCl_2_ to the soluble fractions, resulting in a final concentration of 75 mM. The sample was mixed gently by pipetting and incubated at 25 °C for 2 min before being centrifuged at 16,000 *g* for 2 min. The supernatant was carefully removed, and the precipitate was re-suspended in buffer B12 for washing by gentle pipetting. After performing five washing steps, the precipitate was re-suspended in a cleavage buffer B13 (20 mM Bis-Tris, 75 mM EGTA, pH 6.2) and incubated at 25 °C for 24 h. Following this step, CaCl_2_ was added as before to a final concentration of 75 mM to pellet the RTX tag. The sample was mixed by pipetting and allowed to sit at 25 °C for 2 min before being centrifuged at 16,000 *g* for 2 min. Finally, the soluble fraction was collected by centrifugation.

For the Annexin B1-mediated protein purification, the protocol was modified from a previous report [46]. Harvested cell pellets were first re-suspended in buffer B14 (phosphate buffered saline containing 10 mM Na_2_HPO_4_, 2 mM NaH_2_PO_4_, 137 mM NaCl, 2.7 mM KCl, pH 7.4) containing 1 mM phenylmethanesulfonyl fluoride and 1 mg/mL sodium deoxycholate, and then disrupted by sonication on ice. The soluble fractions were isolated from the cell lysate by centrifugation at 15,000 *g* for 20 min at 4 °C. The precipitation of the Annexin B1 fusion proteins was initiated by adding 2 M CaCl_2_ to the soluble fractions, resulting in a final concentration of 20 mM. The sample was mixed gently by pipetting and incubated at 4 °C for 2 h, followed by centrifugation at 12,000 *g* for 20 min. The supernatant was carefully removed, and the precipitate was re-suspended in a cleavage buffer B15 (20 mM Bis-Tris, 20 mM EDTA, pH 6.2) using a pipette. The sample was incubated at 4 °C for 30 min to redissolve the precipitate and then incubated at 25 °C for 24 h to initiate the intein-mediated cleavage reaction. After that, CaCl_2_ was added as before to a final concentration of 20 mM to pellet the Annexin B1 tag. The sample was mixed by pipetting and incubated at 4 °C for 2 h, then centrifuged at 12,000 *g* for 20 min. Finally, the soluble fraction was collected by centrifugation.

All protein samples were analyzed by 12% SDS-PAGE, SurePage™ 4–20% Bis-Tris SDS-PAGE (GenScript), or 4–12% NuPAGE® Bis-Tris SDS-PAGE (Invitrogen), followed by staining with Coomassie Brilliant Blue R-250. Protein amounts were quantified densitometrically using the software ImageJ (NIH, USA). Bovine serum albumin (BSA) and aprotinin (APR) were used as standards to determine the compositions and protein amounts in all samples.

All buffers used in this study can be found in Additional file 2: Table S6.

### Activity assays

The xylanase activity was determined by measuring the amount of reducing sugar released using the 3,5-dinitrosalicylic acid (DNS) method [50]. Xylose was used as a standard. The reaction mixtures containing 0.5% (W/V) of xylan in buffer B6 (50 mM sodium phosphate, pH 7.0) were incubated at 55 °C for 15 min. One unit of enzyme activity was defined as the amount of enzyme that produced 1 μmol of reducing sugar per minute.

The receptor binding activity of hGH and LCB3 was determined by Biolayer interferometry (BLI). BLI binding data were collected using an Octet RED96 (ForteBio) and processed using the instrument’s integrated software. Growth hormone receptor and SARS-CoV-2 spike protein were individually immobilized to the AR2G Biosensors as described in ForteBio’s technical notes. The baseline was obtained by incubating in buffer B7 (0.1% BSA, 0.02% Tween-20 in 10 mM phosphate buffered saline (PBS), pH 7.4) for 240 s. The association and dissociation steps were performed for 600 s in buffer B7, respectively.

The activity of SpyCatcherΔN-ELP-SpyCatcherΔN was verified by the Spy chemistry-enabled covalent reconstitution [34] and determined by SDS-PAGE. SpyCatcherΔN-ELP-SpyCatcherΔN and LCB3-SpyTag were individually diluted in PBS to 20 μM and 120 μM, respectively. Next, equal volumes of LCB3-SpyTag were individually mixed with three purified SpyCatcherΔN-ELP-SpyCatcherΔN samples (with a molar ratio of the SpyCatcherΔN domain and LCB3-SpyTag of 1:3). The complexes were incubated at 25 °C for 3 h.

### Protein purification by two steps of chromatography

All the four target proteins and peptides were further purified by a two-step chromatography as previously described [26, 27] using an ÄKTA™ pure protein purification system (GE Healthcare, USA). Corresponding resins and buffers, as listed in Additional file 2: Table S4 and Table S6, were utilized for the purification process.

### Reversed-phase HPLC analyses

The three purified target proteins and peptides, hGH, LCB3, and xylanase, were analyzed by reversed-phase HPLC as previously described [27]. However, for the scaffold protein SpyCatcherΔN-ELP-SpyCatcherΔN, the analysis method was modified from the previous report [27]. Briefly, SpyCatcherΔN-ELP-SpyCatcherΔN was analyzed using an Agilent Technologies 1260 system with a C-18 column (5 μm, 4.6 × 150 mm, Agilent, USA). It was eluted with 1 mL/min using a linear gradient from 5 to 95% in mobile phase B (0.1% trifluoroacetic acid in acetonitrile) at 30 °C for 12 min. Detection was achieved by monitoring the UV absorbance at 280 nm.

### Laser scanning confocal microscopic (LSCM) analyses

The LSCM analyses used in this work were adapted from a previous report [51]. Briefly, cells expressing mCherry or MpA-mCherry were cultured at 18 °C for 24 h after 0.2 mM IPTG induction. After harvesting, the cells were fixed with 4% paraformaldehyde and subsequently imaged at 561 nm using a Nikon A1 confocal microscope (Nikon, New York, NY, USA).

### Electronic supplementary material

Below is the link to the electronic supplementary material.

**Additional file 1: Figure S1.** SDS-PAGE analysis of the purification of hGH by three salt-inducible peptides with three high-salt conditions. **Figure S2.** Binding of hGH to the hGH receptor monitored with BLI. **Figure S3.** SDS-PAGE analysis of the redissolving process of the MpA-*Mtu* ΔI-CM-hGH fusion protein. **Figure S4.** Intracellular localization of MpA-mCherry in *E. coli* BL21 (DE3) cells. **Figure S5.** SDS-PAGE analysis of the redissolving process of the MpA-mCherry fusion protein. **Figure S6.** Purification of LCB3/SpyCatcherΔN-ELP-SpyCatcherΔN/xylanase with 3 M NaCl. **Figure S7.** Purification of LCB3/SpyCatcherΔN-ELP-SpyCatcherΔN/xylanase with 0.7 M (NH_4_)_2_SO_4_. **Figure S8.** Binding of LCB3 to the SARS-CoV-2 spike receptor monitored with BLI. **Figure S9.** SDS-PAGE analysis of the Spy chemistry-enabled covalent reconstitution between SpyCatcherΔN-ELP-SpyCatcherΔN and LCB3-SpyTag. **Figure S10.** Enzyme activities of icSAT-purified xylanase. **Figure S11.** SDS-PAGE analysis of the calcium-inducible tags mediated-protein purifications. **Figure S12.** Ion exchange and size exclusion chromatograms using ÄKTA for the purification of target proteins and peptides. **Figure S13.** RP-HPLC characterization of purified target proteins and peptides

**Additional file 2: Table S1.** Comparison of the affinity chromatographic systems. **Table S2.** A comparative study on the different salt-inducible peptides with three salts for purifing hGH. **Table S3.** Calcium-inducible tags mediated recombinant protein expression and purification. **Table S4.** Resin and pH used in the IEC and SEC steps for purification of the target proteins and peptides. **Table S5.** Primers used in this study. **Table S6.** The buffers used in this study

## Data Availability

All data on which the conclusions of this manuscript rely are included in the manuscript and the additional files.

## Abbreviations

BLI: Biolayer interferometry
CP: Precipitate of intein-mediated cleavage
CS: Supernatant of intein-mediated cleavage
cSAT: Cleavable self-aggregating tag
*E. coli*: *Escherichia coli*
ELPs: Elastin-like polypeptides
EP: Precipitate of the cell lysate after expression
ES: Supernatant of the cell lysate after expression
ESP: Precipitate of salt-induced aggregation
ESS: Supernatant of salt-induced aggregation
HCPs: Host cell proteins
hGH: Human growth hormone
icSAT: Inducible and cleavable self-aggregating tag
IEC: Ion-exchange chromatography
*K*_D_: Dissociation constants
MW: The molecular weights
OD_600_: Optical density at 600 nm
pI: Isoelectric point
RTX: Repeat-in-toxin domain
SAP: Self-assembling peptide
SARS-CoV-2: Severe acute respiratory syndrome coronavirus 2
SEC: Size-exclusion chromatography
