# Supplementary material for: A novel protein purification scheme based on salt inducible self-assembling peptides
Source: Microb Cell Fact. 2023 Oct 30;22:224. doi: 10.1186/s12934-023-02229-5 (PMC10614350; doi:10.1186/s12934-023-02229-5)
Supplement: Supplementary file 1 — Additional file 1: Figure S1. SDS-PAGE analysis of the purification of hGH by three salt-inducible peptides with three high-salt conditions. Figure S2. Binding of hGH to the hGH receptor monitored with BLI. Figure S3. SDS-PAGE analysis of the redissolving process of the MpA-Mtu ΔI-CM-hGH fusion protein. Figure S4. Intracellular localization of MpA-mCherry in E. coli BL21 (DE3) cells. Figure S5. SDS-PAGE analysis of the redissolving process of the MpA-mCherry fusion protein. Figure S6. Purification of LCB3/SpyCatcherΔN-ELP-SpyCatcherΔN/xylanase with 3 M NaCl. Figure S7. Purification of LCB3/SpyCatcherΔN-ELP-SpyCatcherΔN/xylanase with 0.7 M (NH4)2SO4. Figure S8. Binding of LCB3 to the SARS-CoV-2 spike receptor monitored with BLI. Figure S9. SDS-PAGE analysis of the Spy chemistry-enabled covalent reconstitution between SpyCatcherΔN-ELP-SpyCatcherΔN and LCB3-SpyTag. Figure S10. Enzyme activities of icSAT-purified xylanase. Figure S11. SDS-PAGE analysis of the calcium-inducible tags mediated-protein purifications. Figure S12. Ion exchange and size exclusion chromatograms using ÄKTA for the purification of target proteins and peptides. Figure S13. RP-HPLC characterization of purified target proteins and peptides [file 12934_2023_2229_MOESM1_ESM.docx]

Additional file 1

Figures S1-S13


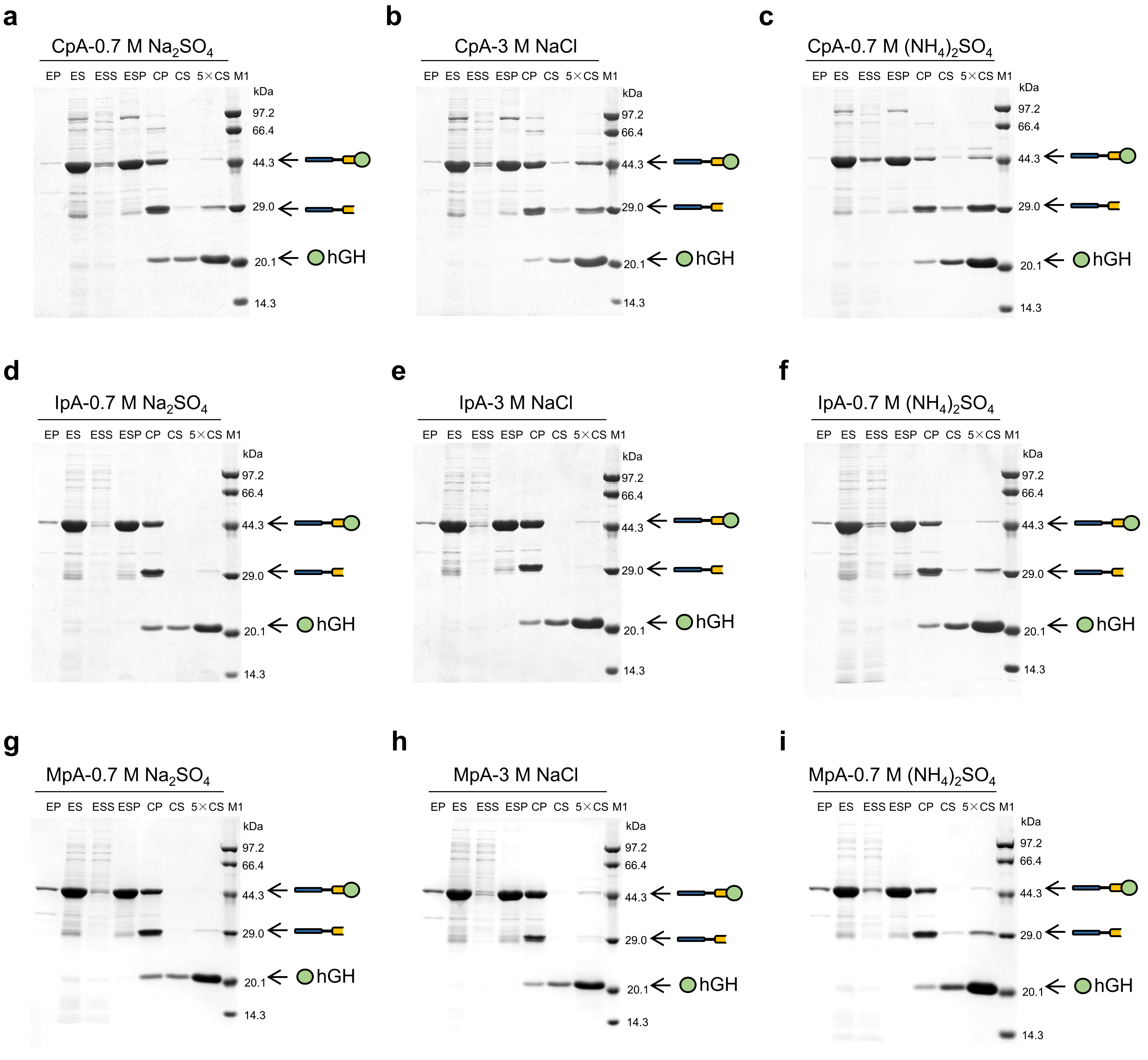


**Fig. S1** SDS-PAGE analysis of the purification of hGH by three salt-inducible peptides with three high-salt conditions. EP: precipitate of the cell lysate after expression, ES: supernatant of the cell lysate after expression, ESS, supernatant of salt-induced aggregation, ESP: precipitate of salt-induced aggregation, CP: precipitate of intein-mediated cleavage, CS and 5×CS: supernatant of intein-mediated cleavage, the 5×CS was loaded at 5 times the amount of the CS. Referring to the quantification results obtained from the CS lane, no notable discrepancy was noticed in the hGH purity between the IpA and MpA variations (data not shown). To conduct a more precise comparison between the two variants, we set up a 5×CS lane, which uncovered the existence of visible impurity bands. M1: protein marker. Purification of hGH from the fusion protein. CpA-*Mtu* ΔI-CM-hGH with a high-salt condition of 0.7 M Na_2_SO_4_ (**a**), 3 M NaCl (**b**), or 0.7 M (NH_4_)_2_SO_4_ (**c**); purification of hGH from the fusion protein IpA-*Mtu* ΔI-CM-hGH with a high-salt condition of 0.7 M Na_2_SO_4_ (**d**), 3 M NaCl (**e**), or 0.7 M (NH_4_)_2_SO_4_ (**f**); purification of hGH from the fusion protein MpA-*Mtu* ΔI-CM-hGH with a high-salt condition of 0.7 M Na_2_SO_4_ (**g**), 3 M NaCl (**h**), or 0.7 M (NH_4_)_2_SO_4_ (**i**).


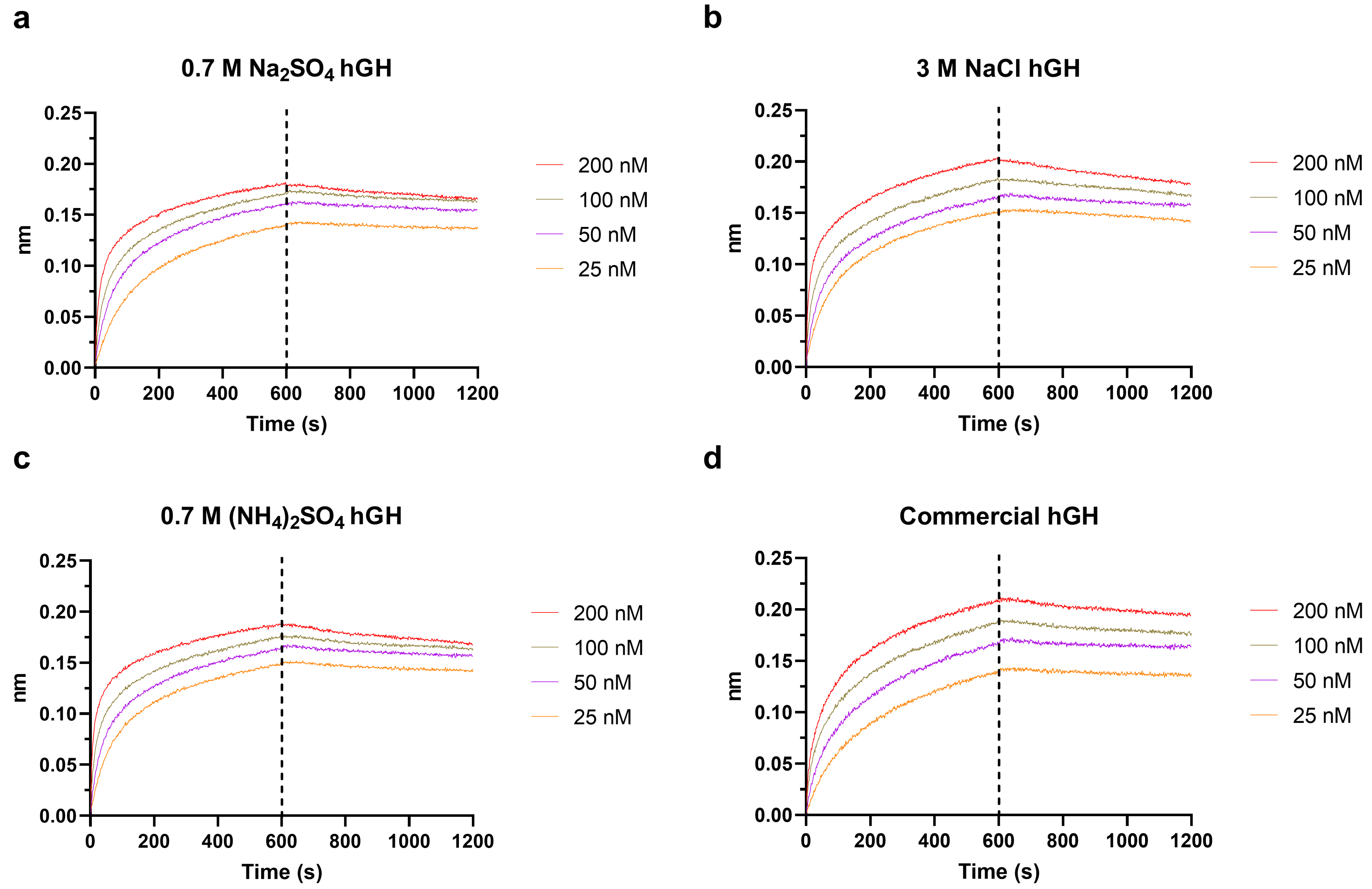


**Fig. S2** Binding of hGH to the hGH receptor monitored with BLI. **a** icSAT-purified hGH with 0.7 M Na_2_SO_4_. **b** icSAT-purified hGH with 3 M NaCl. **c** icSAT-purified hGH with 0.7 M (NH_4_)_2_SO_4_. **d** Commercial hGH.

**
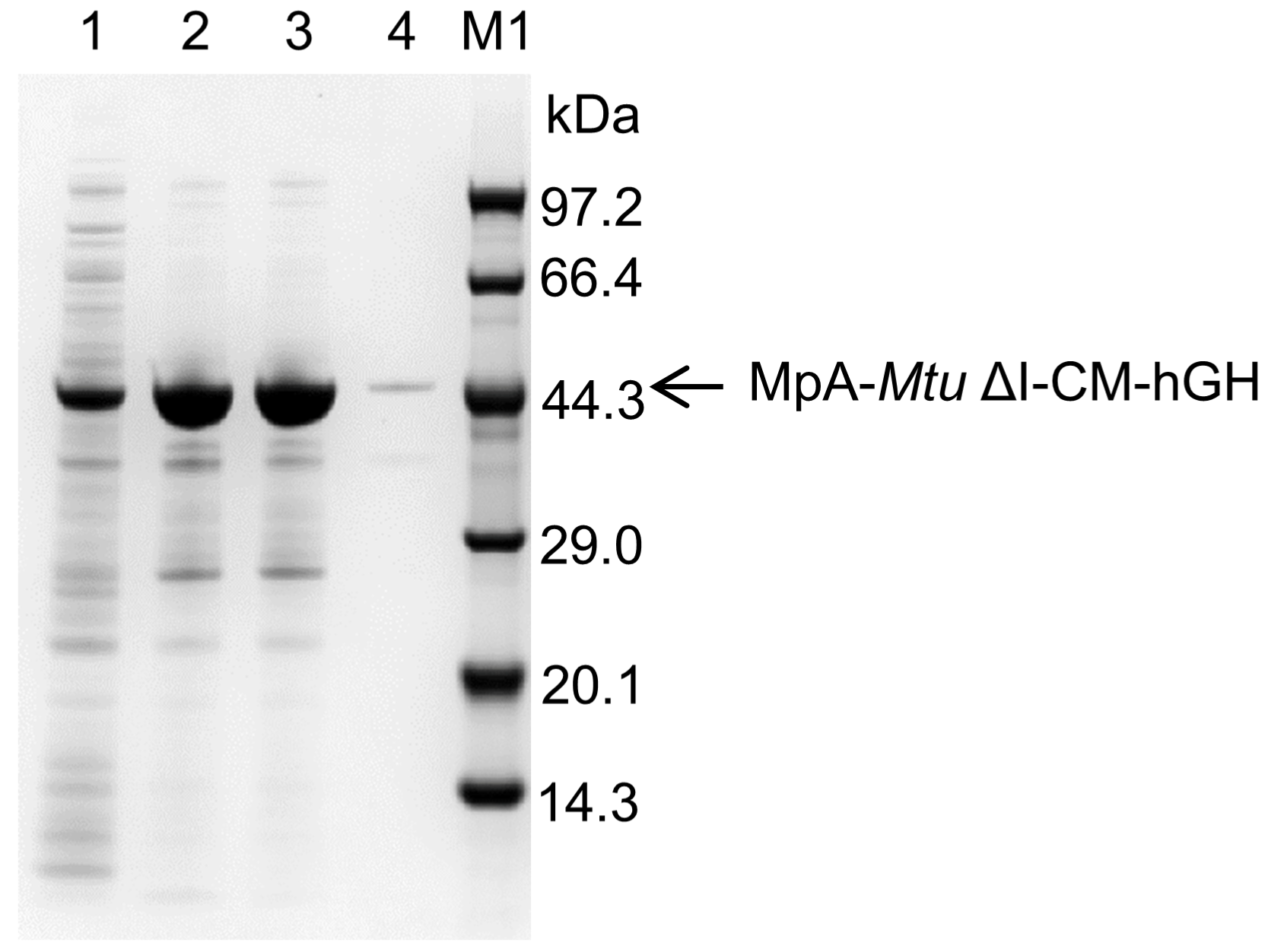
**

**Fig. S3** SDS-PAGE analysis of the redissolving process of the MpA-*Mtu* ΔI-CM-hGH fusion protein. Lane 1: supernatant of the cell lysate, lane 2: precipitate of the cell lysate, lane 3: supernatant of the redissolving product, lane 4: precipitate of the redissolving product, M1: protein marker.


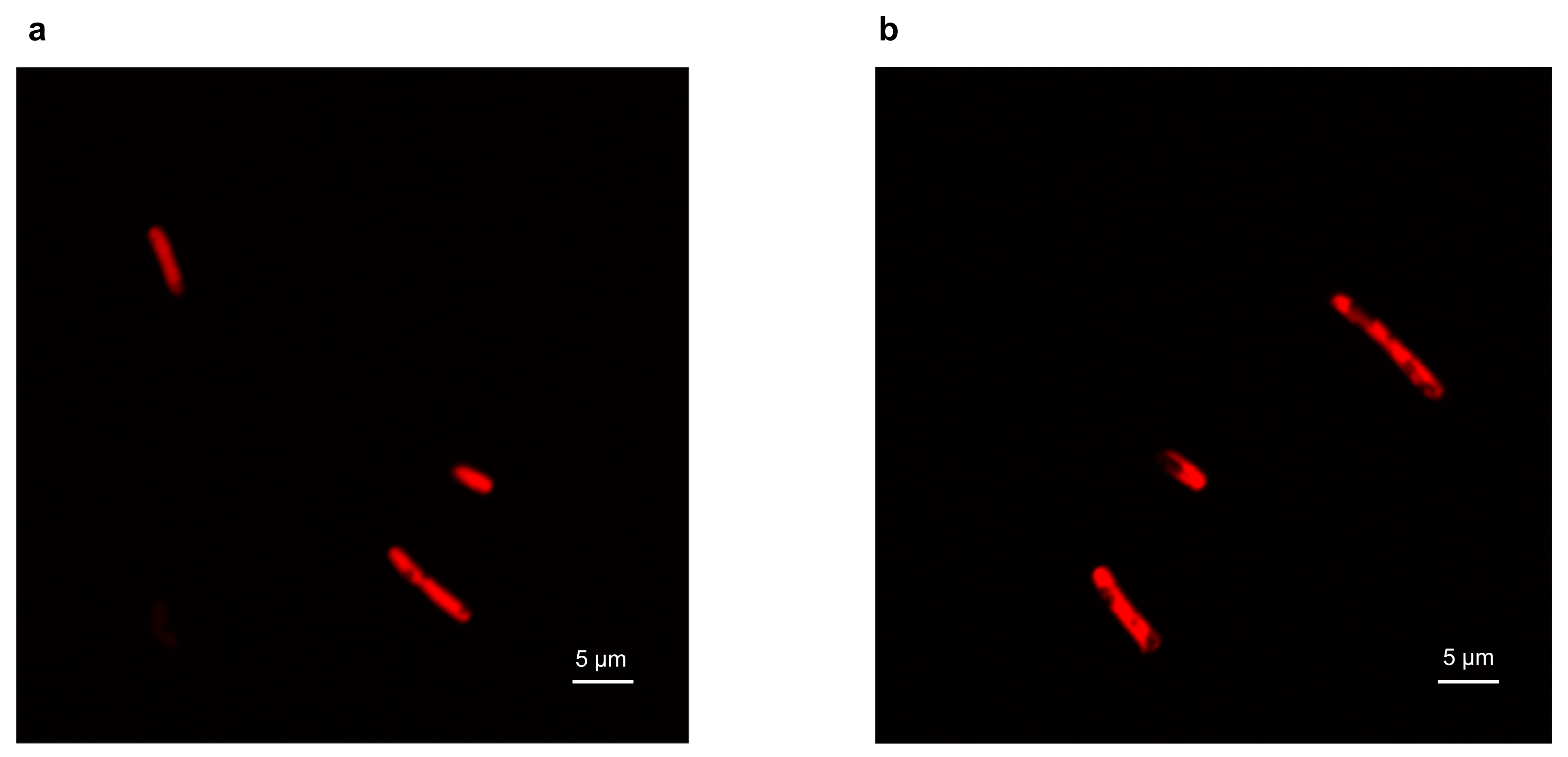


**Fig. S4** Intracellular localization of MpA-mCherry in *E. coli* BL21 (DE3) cells. **a** mCherry. **b** MpA-mCherry.

**
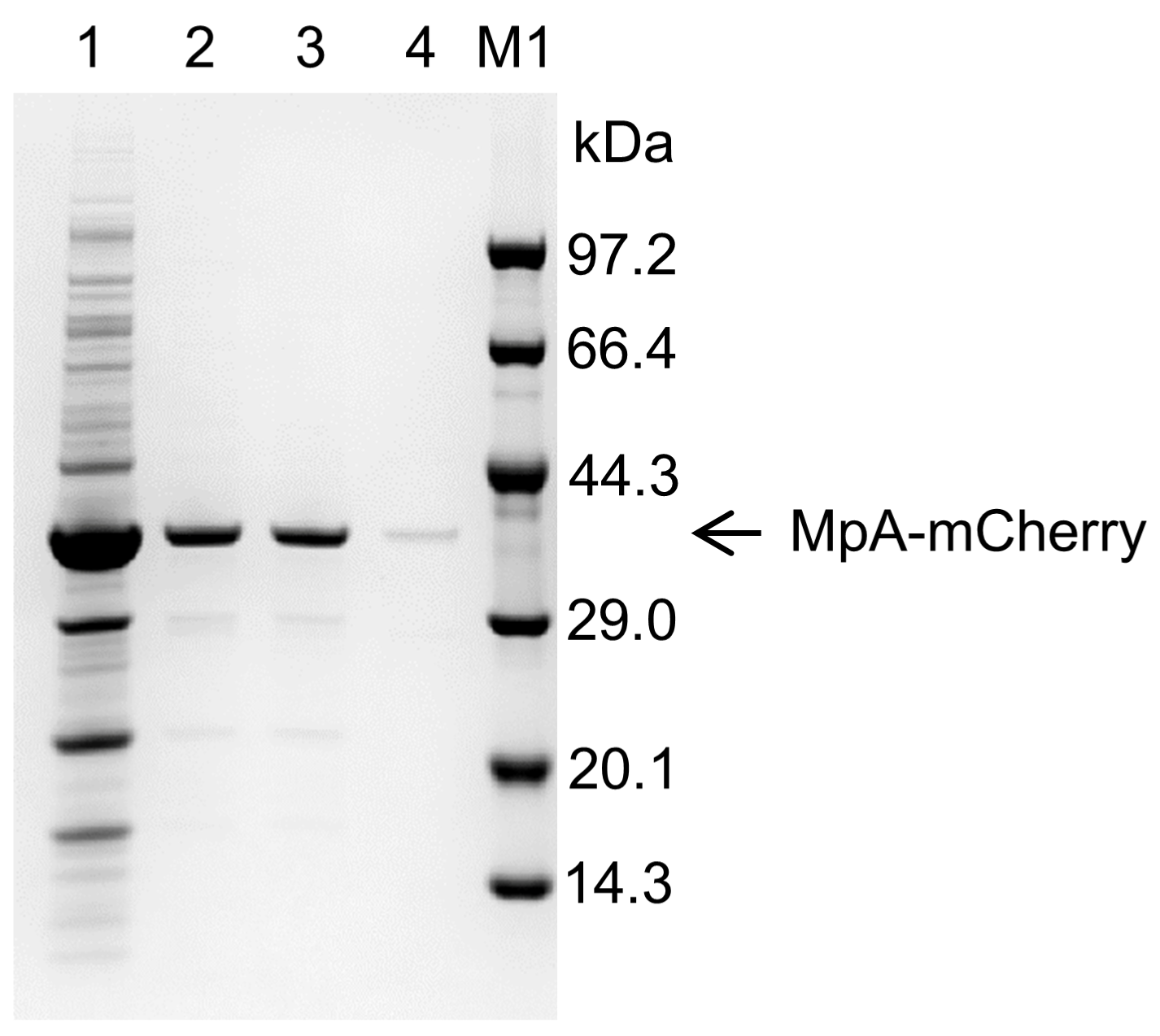
**

**Fig. S5** SDS-PAGE analysis of the redissolving process of the MpA-mCherry fusion protein. Lane 1: supernatant of the cell lysate, lane 2: precipitate of the cell lysate, lane 3: supernatant of the redissolving product, lane 4: precipitate of the redissolving product, M1: protein marker.

**
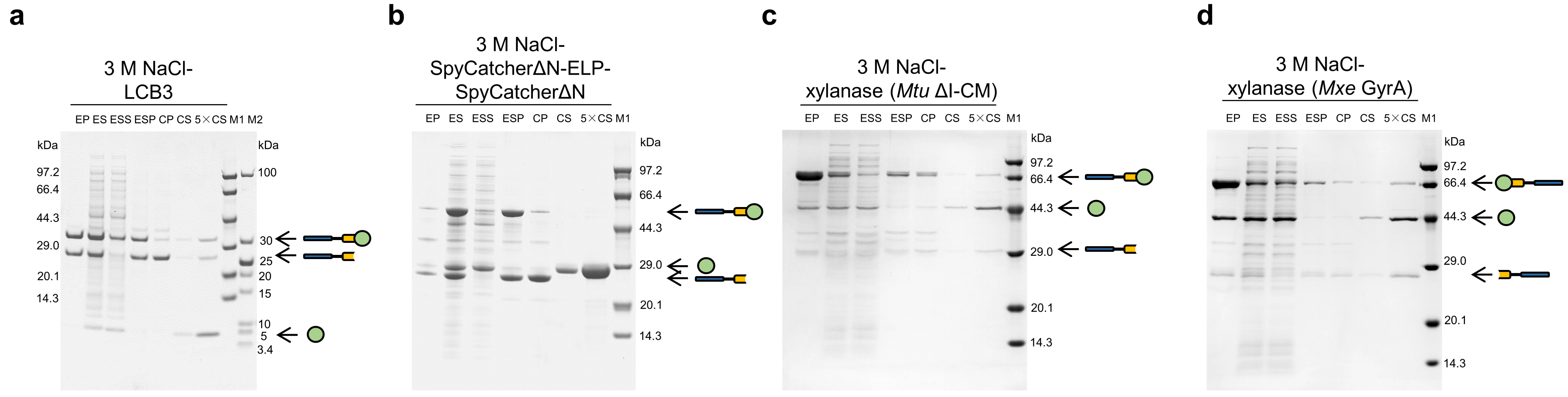
**

**Fig. S6** Purification of LCB3/SpyCatcherΔN-ELP-SpyCatcherΔN/xylanase with 3 M NaCl. M1 and M2: protein marker. EP: precipitate of the cell lysate after expression, ES: supernatant of the cell lysate after expression, ESS, supernatant of salt-induced aggregation, ESP: precipitate of salt-induced aggregation, CP: precipitate of intein-mediated cleavage, CS and 5×CS: supernatant of intein-mediated cleavage, the 5×CS was loaded at 5 times the amount of the CS. **a** LCB3. **b** SpyCatcherΔN-ELP-SpyCatcherΔN. **c-d** Purification results for xylanase from the two fusion proteins, MpA-*Mtu* ΔI-CM-xylanase (**c**) and xylanase-*Mxe* GyrA-MpA (**d**), individually.

**
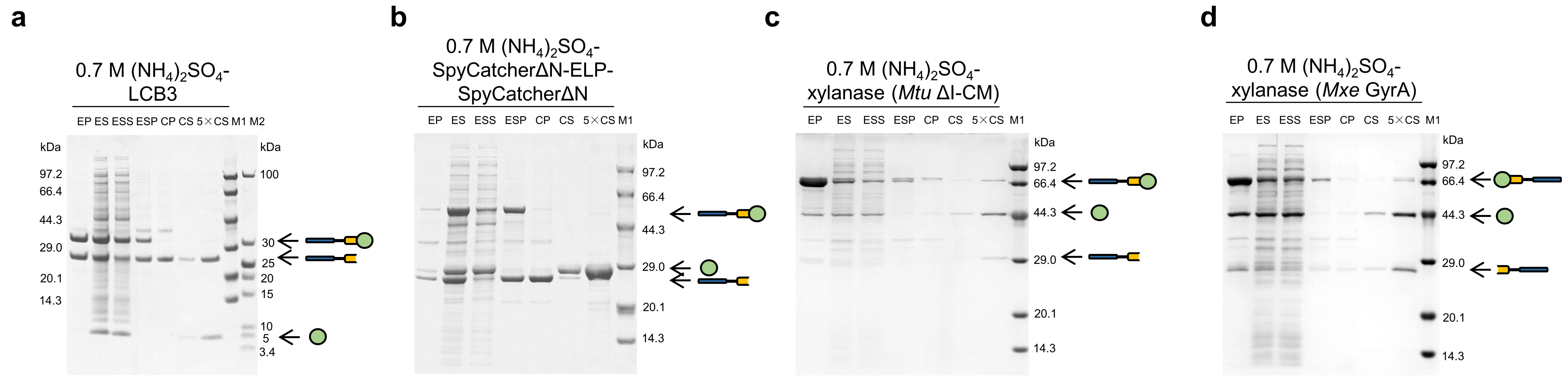
**

**Fig. S7** Purification of LCB3/SpyCatcherΔN-ELP-SpyCatcherΔN/xylanase with 0.7 M (NH_4_)_2_SO_4_. M1 and M2: protein marker. EP: precipitate of the cell lysate after expression, ES: supernatant of the cell lysate after expression, ESS, supernatant of salt-induced aggregation, ESP: precipitate of salt-induced aggregation, CP: precipitate of intein-mediated cleavage, CS and 5×CS: supernatant of intein-mediated cleavage, the 5×CS was loaded at 5 times the amount of the CS. **a** LCB3. **b** SpyCatcherΔN-ELP-SpyCatcherΔN. **c-d** Purification results for xylanase from the two fusion proteins, MpA-*Mtu* ΔI-CM-xylanase (**c**) and xylanase-*Mxe* GyrA-MpA (**d**), individually.

**
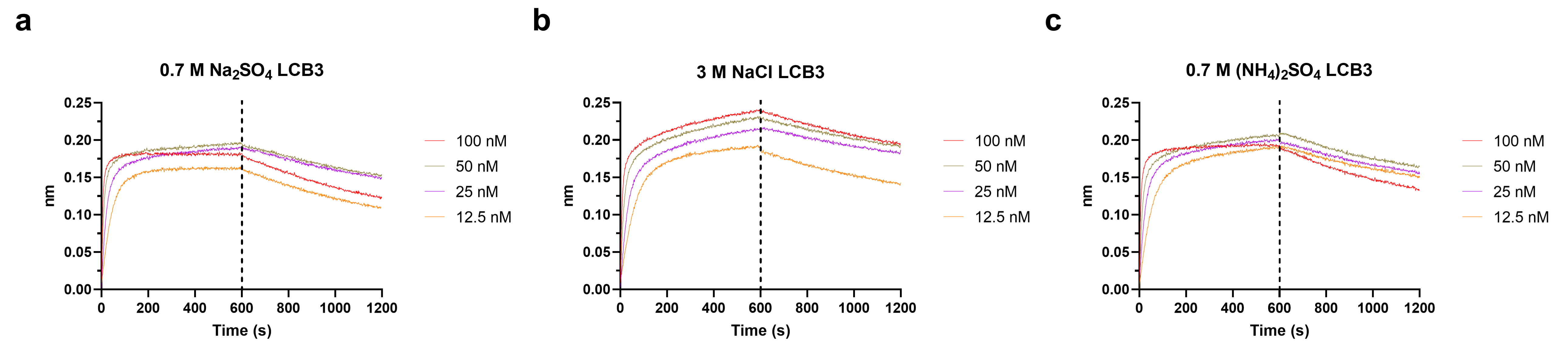
**

**Fig. S8** Binding of LCB3 to the SARS-CoV-2 spike receptor monitored with BLI. **a** icSAT-purified LCB3 with 0.7 M Na_2_SO_4_. **b** icSAT-purified LCB3 with 3 M NaCl. **c** icSAT-purified LCB3 with 0.7 M (NH_4_)_2_SO_4_.

**
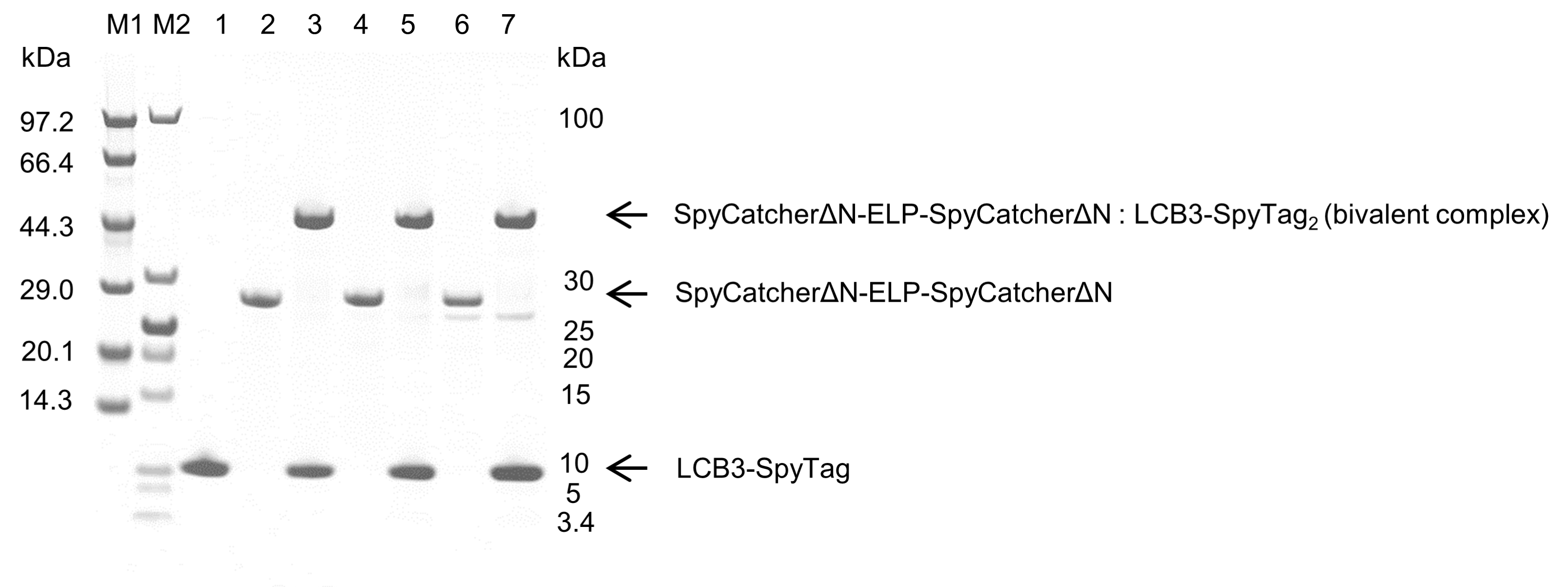
**

**Fig. S9** SDS-PAGE analysis of the Spy chemistry-enabled covalent reconstitution between SpyCatcherΔN-ELP-SpyCatcherΔN and LCB3-SpyTag. LCB3-SpyTag (Lane 1); the purified SpyCatcherΔN-ELP-SpyCatcherΔN with 3 M NaCl (Lane 2), 0.7 M Na_2_SO_4_ (Lane 4) and 0.7 M (NH_4_)_2_SO_4_ (Lane 6); the Spy chemistry-enabled covalent reconstitution reaction products for three groups, *i.e.* 3 M NaCl (Lane 3), 0.7 M Na_2_SO_4_ (Lane 5) and 0.7 M (NH_4_)_2_SO_4_ (Lane 7).

**
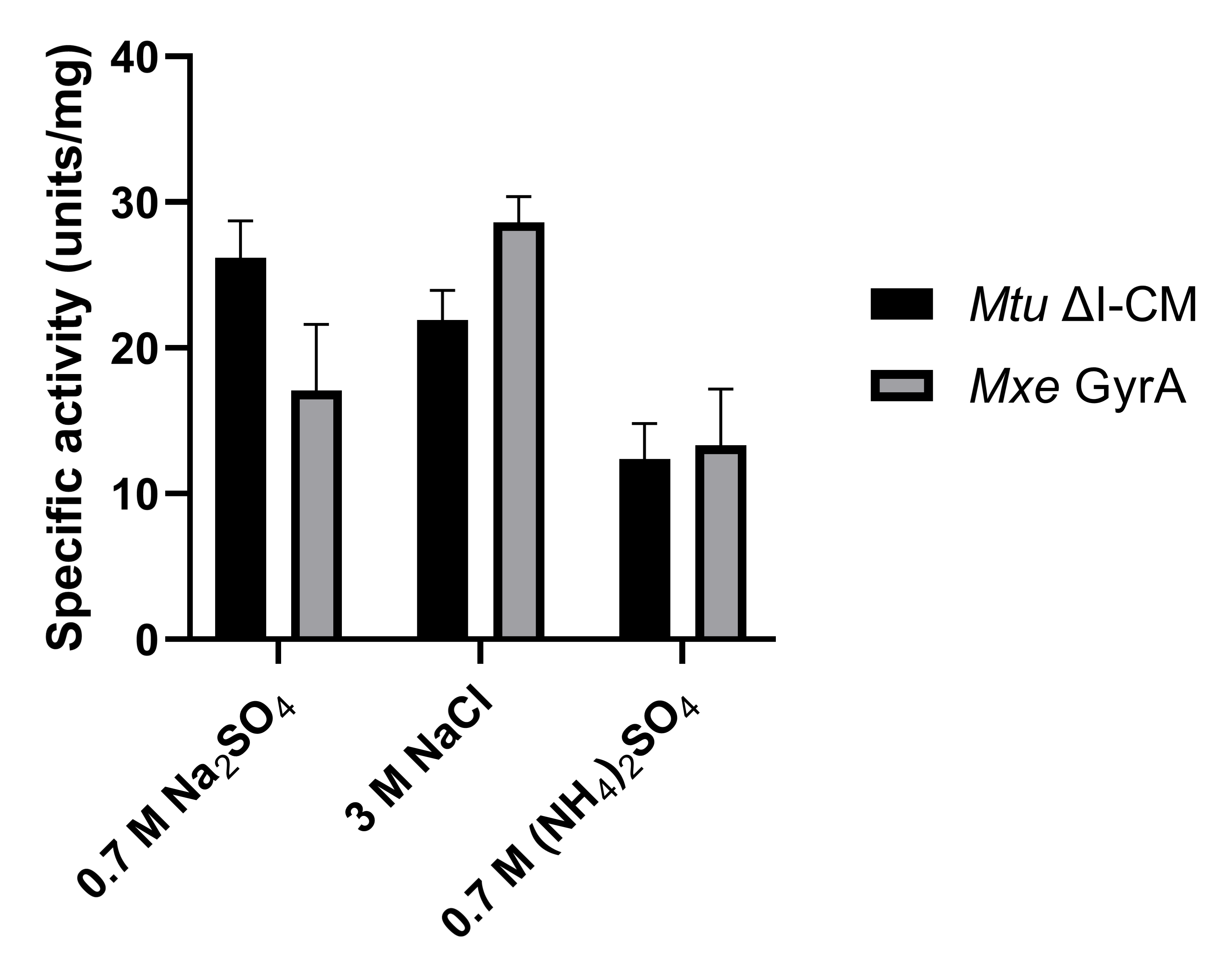
**

**Fig. S10** Enzyme activities of icSAT-purified xylanase.

**
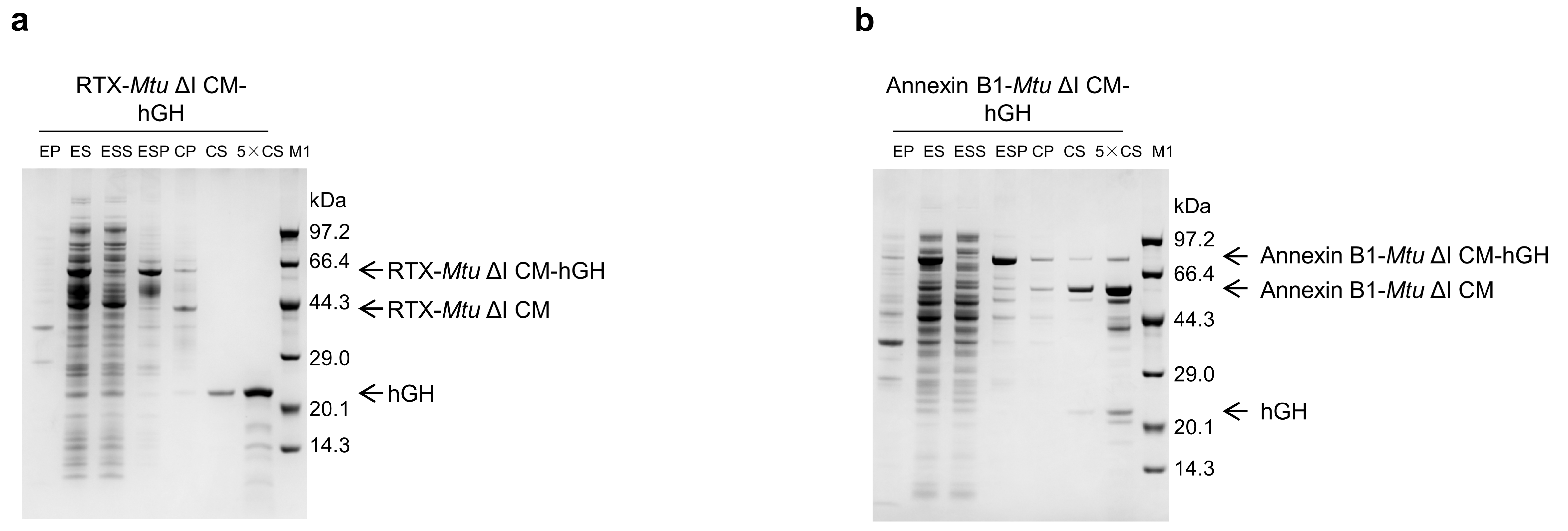
**

**Fig. S11** SDS-PAGE analysis of the calcium-inducible tags mediated-protein purifications. **a** hGH purified from the fusion protein RTX-*Mtu* ΔI CM-hGH with 75 mM CaCl_2_. **b** hGH purified from the fusion protein Annexin B1-*Mtu* ΔI CM-hGH with 20 mM CaCl_2_.

**
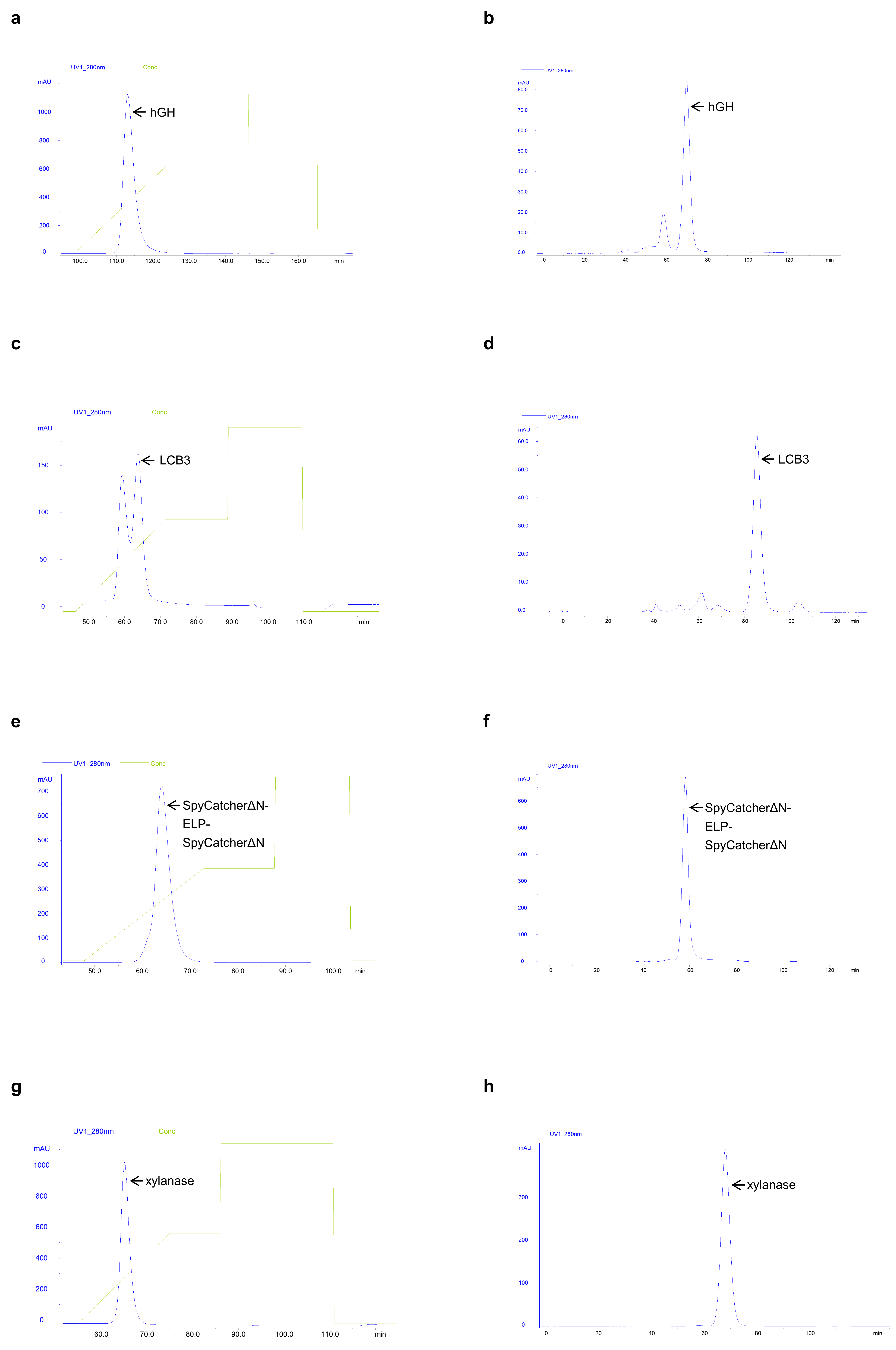
**

**Fig. S12** Ion exchange and size exclusion chromatograms using ÄKTA for the purification of target proteins and peptides. The anion exchange resin Capto Q column chromatogram of hGH (**a**), LCB3 (**c**) and SpyCatcherΔN-ELP-SpyCatcherΔN (**e**); the anion exchange resin Capto S column chromatogram of xylanase (**g**); the HiLoad 16/600 Superdex 75 column chromatogram of hGH (**b**), LCB3 (**d**), SpyCatcherΔN-ELP-SpyCatcherΔN (**f**) and xylanase (**h**).


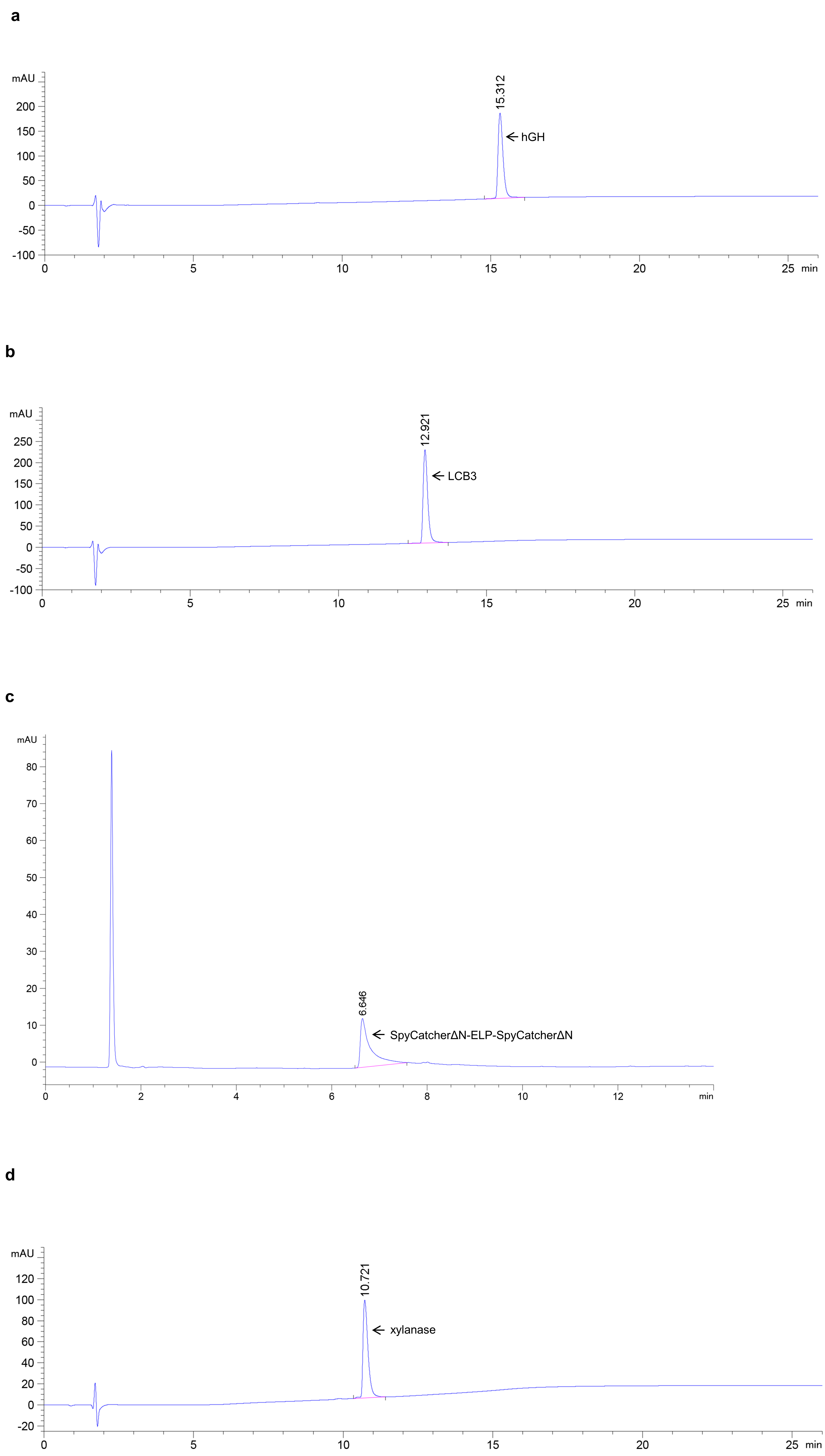


**Fig. S13** RP-HPLC characterization of purified target proteins and peptides. **a** hGH. **b** LCB3. **c** SpyCatcherΔN-ELP-SpyCatcherΔN. **d** xylanase.
