# Supplementary material for: A novel protein purification scheme based on salt inducible self-assembling peptides
Source: Microb Cell Fact. 2023 Oct 30;22:224. doi: 10.1186/s12934-023-02229-5 (PMC10614350; doi:10.1186/s12934-023-02229-5)
Supplement: Supplementary file 2 — Additional file 2: Table S1. Comparison of the affinity chromatographic systems. Table S2. A comparative study on the different salt-inducible peptides with three salts for purifing hGH. Table S3. Calcium-inducible tags mediated recombinant protein expression and purification. Table S4. Resin and pH used in the IEC and SEC steps for purification of the target proteins and peptides. Table S5. Primers used in this study. Table S6. The buffers used in this study [file 12934_2023_2229_MOESM2_ESM.docx]

Additional file 2

Tables S1-S6

**Table S1** Comparison of the affinity chromatographic systems.

| Tag | Resin | Capacity (mg/mL) [1, 2] | Estimated cost of resin | Purity of target protein (%) |
| --- | --- | --- | --- | --- |
| His | Ni-NTA | 20-40 | $15 /mL (Qiagen) | > 80 [3] |
| GST | Glutathione | 10 | $54 /mL (Sigma) | > 80 [4] |
| MBP | Amylose | 6-10 | $13 /mL (Biolabs) | 75 [5] |
| CBP | Calmodulin | 1.5 | $227 /mL (Sigma) | > 95 [6] |
| FLAG | M2 antibody | 0.6 | $1,020 /mL (Sigma) | > 95 [7] |

**Table S2** A comparative study on the different salt-inducible peptides with three salts for purifing hGH.

| Salt-inducible peptide | Fusion protein | Yield of soluble fusion protein (mg/L) | Solubility ratio of fusion protein^a^（%） | 0.7 M Na_2_SO_4_ | | | | 3 M NaCl | | | | 0.7 M (NH_4_)_2_SO_4_ | | | | |
| --- | --- | --- | --- | --- | --- | --- | --- | --- | --- | --- | --- | --- | --- | --- | --- | --- |
|  |  |  |  | Aggregation efficiency^b^ (%) | Cleavage efficiency^c^ (%) | Yield^d^ (mg/L) | Purity^e^ (%) | Aggregation efficiency^b^ (%) | Cleavage efficiency^c^ (%) | Yield^d^ (mg/L) | Purity^e^ (%) | Aggregation efficiency^b^ (%) | Cleavage efficiency^c^ (%) | Yield^d^ (mg/L) | Purity^e^ (%) |  |
| CpA | CpA-*Mtu* ΔI-CM-hGH | 459 ± 38 | 98 ± 1 | 88 ± 1 | 59 ± 1 | 63 ± 18 | 79 ± 1 | 91 ± 2 | 47 ± 7 | 50 ± 15 | 67 ± 9 | 77 ± 4 | 72 ± 6 | 102 ± 7 | 55 ± 3 |  |
| IpA | IpA-*Mtu* ΔI-CM-hGH | 505 ± 12 | 93 ± 1 | 97 ± 1 | 59 ± 1 | 55 ± 4 | 95 ± 1 | 97 ± 2 | 37 ± 6 | 71 ± 8 | 94 ± 1 | 89 ± 1 | 66 ± 3 | 112 ± 17 | 84 ± 1 |  |
| MpA | MpA-*Mtu* ΔI-CM-hGH | 611 ± 32 | 89 ± 6 | 95 ± 1 | 56 ± 7 | 87 ± 6 | 97 ± 1 | 95 ± 2 | 39 ± 9 | 81 ± 12 | 94 ± 7 | 89 ± 6 | 65 ± 11 | 127 ± 16 | 88 ± 7 |  |

^a^Solubility ratio is defined as the mass ratio of the soluble fusion protein to the total fusion protein in the cell lysate. ^b^Aggregation efficiency is the ratio of the mass of insoluble fusion protein precipitated by salt to the total mass of fusion protein (both soluble and insoluble) treated with salt-induced aggregation. ^c^Cleavage efficiency is the ratio of the mass of cleaved protein aggregate to the total aggregate before cleavage. ^d^Yield of hGH after intein-mediated cleavage per liter of culture. ^e^Purity is calculated as the mass ratio of hGH to total proteins in the supernatant after intein-mediated cleavage cleavage, estimated using densitometry analysis software ImageJ.

**Table S3.** Calcium-inducible tags mediated recombinant protein expression and purification.

| Tags | Fusion protein | OD_600_^a^ | Yield of soluble fusion proteins (mg/L) | Purified hGH | |
| --- | --- | --- | --- | --- | --- |
|  |  |  |  | Yield (mg/L)^b^ | Purity (%)^c^ |
| RTX | RTX-*Mtu* ΔI-CM-hGH | 1.33 ± 0.08 | 28 ± 1 | 6 ± 1 | 84 ± 3 |
| Annexin B1 | Annexin B1-*Mtu* ΔI-CM-hGH | 3.35 ± 0.02 | 90 ± 12 | 8 ± 3 | 18 ± 6 |

^a^The OD_600_ (optical density at 600 nm) of strains was measured after 24 hours’ incubation. ^b^Yield of hGH after intein-mediated cleavage per liter of culture. ^c^Purity is calculated as the mass ratio of hGH to total proteins in the supernatant after intein-mediated cleavage cleavage, estimated using densitometry analysis software ImageJ.

**Table S4.** Resin and pH used in the IEC and SEC steps for purification of the target proteins and peptides.

| Target protein | IEC | | SEC | |
| --- | --- | --- | --- | --- |
|  | Isoelectric point^a^ | Resin, pH | Molecular weight (kDa)^b^ | Resin, pH |
| hGH | 5.3 | Capto Q, 7.2 | 22.1 | Superdex75, 7.2 |
| LCB3 | 4.9 | Capto Q, 7.2 | 7.7 | Superdex75, 7.2 |
| SpyCatcherΔN-ELP-SpyCatcherΔN | 4.9 | Capto Q, 7.2 | 26.2 | Superdex75, 7.2 |
| xylanase | 8.2 | Capto S, 7.0 | 45.9 | Superdex75, 7.2 |

^a, b^The theoretical isoelectric point and molecular weight of the four target proteins and peptides were calculated by Compute pI/Mw tool (<https://web.expasy.org/compute_pi/>).

**Table S5** Primers used in this study.

| Primer name | Nucleotide sequence^a^ | Description |
| --- | --- | --- |
| MpA-PT-1 | 5′-GGGAATTCCATATGAAACAACTGGAAGACAAGATTGAAGAACTGTTAAGCAAAGCGG-3' (*Nde*I) | Used for synthesis of MpA-PT linker DNA fragment for construction of pET30a-MpA-*Mtu* ΔI-CM-hGH |
| MpA-PT-2 | 5′-ACAGCAGTTCCTCTATTTTGTCCTCCAGCTGCTTCATCGCCGCTTTGCTTAACAGTTCT-3′ |  |
| MpA-PT-3 | 5′-GACAAAATAGAGGAACTGCTGTCCAAGCCGACCCCACCGACCACGCCAACGCCACCAAC-3′ |  |
| MpA-PT-4 | 5′-GTGCCTTCAGCCAGCGCGAATTCCGGCGTCGGGGTTGGGGTGGTTGGTGGCGTTGGCG-3′ |  |
| GS-MpA-1 | 5′-CGAACGGGTTCGTCAGCCACGCTAAGCTTGGCGGCGGTG-3' | Used for synthesis of GS linker-MpA DNA fragment for construction of pET30a-xylanase-*Mxe* GyrA-MpA |
| GS-MpA-2 | 5′-CTGCTTCATGCTACCGCCGCCACCGGAACCGCCACCGCCAGAACCACCGCCGCCAAGCT-3' |  |
| GS-MpA-3 | 5′-GGCGGTAGCATGAAGCAGCTGGAAGACAAAATTGAGGAACTCCTGTCTAAAGCGGCGAT-3' |  |
| GS-MpA-4 | 5′-TTTAGAGAGCAGCTCTTCGATCTTGTCCTCCAGTTGTTTCATCGCCGCTTTAGACAGGA-3' |  |
| MpA-F | 5′-GGGAATTCCATATGAAACAACTGGAAGACAAGATTGAAG-3′ (*Nde*I) | Used for amplification of MpA-PT linker DNA fragment for construction of pET30a-MpA-*Mtu* ΔI-CM-hGH |
| PT-Mtu-R | 5′-GTGCCTTCAGCCAGCGCGAATTCCGGCGTCGGGGTTGG-3′ |  |
| Mtu-F | 5′-TCGCGCTGGCTGAAGGCAC-3′ | Used for amplification of *Mtu* ΔI-CM-hGH DNA fragment for construction of pET30a-MpA-*Mtu* ΔI-CM-hGH |
| Mtu-hGH-R | 5′-ATCCGCTCGAGTCAGAAACCGCAAGA-3′ (*Xho*I) |  |
| Backbone-F | 5'-CCTTTGAGTGAGCTGATAC-3' | Used for amplification of *lacI*-CpA DNA fragment for construction of pET30a-CpA-*Mtu* ΔI-CM-hGH |
| CpA-R | 5′-CTTCAATCTTGTCTTCCAGTTGTTTGCACATATGTATATCTCCTTCTTAAAGTTAAACA-3′ |  |
| Backbone-R | 5'-CGGTATCAGCTCACTCAAAGG-3' | Used for amplification of CpA-*Mtu* ΔI-CM-hGH-*KanR* DNA fragment for construction of pET30a-CpA-*Mtu* ΔI-CM-hGH |
| CpA-F | 5′-ACTGGAAGACAAGATTGAAGAACTGTTAAGCAAAGCGGCGTGCAAGCAGCTGGAGGACA-3′ |  |
| IpA-F | 5′-ACTGGAAGACAAGATTGAAGAACTGTTAAGCAAAGCGGCGATCAAGCAGCTGGAGGACA-3′ | Used together with Backbone-R for amplification of IpA-*Mtu* ΔI-CM-hGH-*KanR* DNA fragment for construction of pET30a-IpA-*Mtu* ΔI-CM-hGH |
| IpA-R | 5′-CTTCAATCTTGTCTTCCAGTTGTTTGATCATATGTATATCTCCTTCTTAAAGTTAAACA-3′ | Used together with Backbone-F for amplification of *lacI*-IpA DNA fragment for construction of pET30a-IpA-*Mtu* ΔI-CM-hGH |
| Mtu-R | 5′-GTTATGAACCACAACGCCTTCCGCAACCAG-3′ | Used together with Mtu-F for amplification of *Mtu* ΔI-CM DNA fragment for construction of pET30a-MpA-*Mtu* ΔI-CM-LCB3 |
| SpyCatcherΔN-F | 5′-CGGAAGGCGTTGTGGTTCATAACAGCGCCACCCATATTAAATTTTCTA-3′ | Used for amplification of SpyCatcherΔN-ELP-SpyCatcherΔN DNA fragment for construction of pET30a-MpA-*Mtu* ΔI-CM-SpyCatcherΔN-ELP-SpyCatcherΔN |
| SpyCatcherΔN-R | 5′-GCTTTGTTAGCAGCCGGATCTC-3′ |  |
| SpyCatcherΔN-Backbone-F | 5′-GATCCGGCTGCTAACAAAGCCCGAAAGGA-3′ | Used together with Mtu-R for amplification of *KanR-lacI*-MpA-*Mtu* ΔI-CM-1 DNA fragment for construction of pET30a-MpA-*Mtu* ΔI-CM-SpyCatcherΔN-ELP-SpyCatcherΔN |
| LCB3-F | 5′-GCGGAAGGCGTTGTGGTTCATAACAACGATGACGAACTGCACATGCT-3' | Used for amplification of LCB3 DNA fragment for construction of pET30a-MpA-*Mtu* ΔI-CM-LCB3 |
| LCB3-R | 5′-GCTCAGCAGACGTTCCAGCAGT-3' |  |
| LCB3-Backbone-F | 5′-AGAACTGCTGGAACGTCTGCTGAGCTGACTCGAGCACCACCACCACCACCACT-3' | Used for amplification of *KanR-lacI*-MpA *Mtu* ΔI-CM-2 DNA fragment for construction of pET30a-MpA-*Mtu* ΔI-CM-LCB3 |
| LCB3-Backbone-R | 5′-GTGCCTTCAGCCAGCGCGAAT-3' |  |
| Backbone-MpA-F | 5′-GAGATCCGGCTGCTAACAAAGCC-3' | Used for amplification of *KanR-lacI*-MpA-*Mtu* ΔI-CM-3 DNA fragment for construction of pET30a-MpA-*Mtu* ΔI-CM-xylanase |
| Mtu-2-R | 5′-GTTATGAACCACAACGCCTTCCGCA-3' |  |
| xylanase-1-F | 5′-GCGGAAGGCGTTGTGGTTCATAAC-3' | Used for amplification of xylanase-1 DNA fragment for construction of pET30a-MpA-*Mtu* ΔI-CM-xylanase |
| xylanase-1-R | 5′-GCTTTGTTAGCAGCCGGATCT-3' |  |
| GS-MpA-F | 5′-CGAACGGGTTCGTCAGCCACGCTAAGCTTGGCG-3' | Used for amplification of GS linker-MpA DNA fragment for construction of pET30a-xylanase-*Mxe* GyrA-MpA |
| MpA-R | 5′-TTTAGAGAGCAGCTCTTCGATCTTGTCCTCCAGTTGTTTCATCG-3' |  |
| xylanase-Mxe-F | 5′-GATGTACCTGCCGATGCGAATGTGCATCACGGGAGATGCACTAGTTG-3' | Used for amplification of *Mxe* GyrA DNA fragment for construction of pET30a-xylanase-*Mxe* GyrA-MpA |
| Mxe-R | 5′-AGCGTGGCTGACGAACCCGTTCG-3' |  |
| xylanase-2-F | 5′-GTGCACAGCAAAACCCCGGATAT-3' | Used for amplification of xylanase-2 DNA fragment for construction of pET30a-xylanase-*Mxe* GyrA-MpA |
| xylanase-2-R | 5′-CATTCGCATCGGCAGGTACATCGCGAATTTAC-3' |  |
| 30a-F | 5′-ACAAGATCGAAGAGCTGCTCTCTAAATGACTCGAGCACCACCACCACCACCACTGA-3' | Used for amplification of *KanR-lacI* DNA fragment for construction of pET30a-xylanase-*Mxe* GyrA-MpA |
| 30a-R | 5′-CCGGGGTTTTGCTGTGCACAGCCATATGTATATCTCCTTCTTAAAGTTAAACA-3' |  |
| Backbone-2-F | 5′-CCTTTGAGTGAGCTGATACCGCTCG-3' | Used for the amplification of *lacI*-MpA DNA fragment for construction of pET30a-MpA-mCherry |
| mCherry-R | 5′-GCCATGTTGTCTTCTTCACCTTTAGAAACGAATTCCGGCGTCGGGGTTGG-3' |  |
| Backbone-2-R | 5′-CGAGCGGTATCAGCTCACTCAAAG-3' | Used for amplification of mCherry-KanR DNA fragment for construction of pET30a-MpA-mCherry |
| mCherry-F | 5′-CGTTTCTAAAGGTGAAGAAGACAACATGGCTA-3' |  |
| T7-RTX-F | 5′-GTCCGGCGTAGAGGATCGAG-3' | Used for amplification of T7-RTX-MtuN DNA fragment for construction of pET30a-RTX-*Mtu* ΔI-CM-hGH |
| MtuN-R | 5′-CAACATCTTCAATGCGGTGCGTCGTGCCC-3' |  |
| MtuN-F | 5′-ACGCACCGCATTGAAGATGTT-3' | Used for amplification of MtuC-hGH-*ori*-1 DNA fragment for construction of pET30a-RTX-*Mtu* ΔI-CM-hGH |
| T7-R | 5′-CTCGATCCTCTACGCCG-3' |  |
| Annexin B1-F | 5′-GCCTACTGTCGCTCCCTGGT-3' | Used for amplification of Annexin B1-MtuN DNA fragment for construction of pET30a-Annexin B1-*Mtu* ΔI-CM-hGH |
| MtuN-2-R | 5′-CAACATCTTCAATGCGGTGCGTC-3' |  |
| Annexin B1-R | 5′-ACCAGGGAGCGACAGTAGGCCATATGTATATCTCCTTCTTAAAGTTAAACA-3' | Used together with MtuN-F for amplification of MtuC-hGH-*ori*-2 DNA fragment for construction of pET30a-Annexin B1-*Mtu* ΔI-CM-hGH |

^a^The underlined nucleotides indicate restriction sites.

**Table S6** The buffers used in this study.

| Buffer | Description | Components |
| --- | --- | --- |
| buffer B1 | a low salt lysis buffer in icSAT-mediated purification, with an ionic strength^a^ of ~0.011 M | 20 mM Tris-HCl, 1 mM EDTA, pH 8.0 |
| buffer B2 | a high salt buffer in icSAT-mediated purification | 20 mM Tris-HCl, 1.4 M Na_2_SO_4_/1.4 M (NH_4_)_2_SO_4_, 1 mM EDTA, pH 8.0 |
| buffer B3 | a high salt resuspension buffer in icSAT-mediated purification | 20 mM Tris-HCl, 0.7 M Na_2_SO_4_/3 M NaCl/0.7 M (NH_4_)_2_SO_4_, 1 mM EDTA, pH 8.0 |
| buffer B4 | a high salt cleavage buffer of *Mtu* ΔI-CM in icSAT-mediated purification | 20 mM Bis-Tris, 0.7 M Na_2_SO_4_/3 M NaCl/0.7 M (NH_4_)_2_SO_4_, 2.7 mM KCl, 10 mM Na_2_HPO_4_, 1.8 mM KH_2_PO_4_, 2 mM EDTA, pH 6.2 |
| buffer B5 | a high salt cleavage buffer of *Mxe* GyrA in icSAT-mediated purification | 20 mM Tris-HCl, 0.7 M Na_2_SO_4_/3 M NaCl/0.7 M (NH_4_)_2_SO_4_, 1 mM EDTA, 40 mM DTT, pH 8.0 |
| buffer B6 | a buffer for the xylanase assay | 50 mM sodium phosphate, pH 7.0 |
| buffer B7 | a kinetics buffer for biolayer interferometry | 0.1% BSA, 0.02% Tween-20 in 10 mM PBS, pH 7.4 |
| buffer B8 | a lysis buffer with an ionic strength^b^ of ~0.237 M, similar to that of the intracellular environment of *E. coli* | 20 mM Tris-HCl, 225 mM NaCl, 1 mM EDTA, pH 8.0 |
| buffer B9 | a start buffer for Capto Q resin in ion-exchange chromatography | 20 mM Tris-HCl, pH 7.2 |
| buffer B10 | a start buffer for Capto S resin in ion-exchange chromatography | 50 mM sodium phosphate, pH 7.0 |
| buffer B11 | a running buffer in size-exclusion chromatography | 50 mM sodium phosphate, 150 mM NaCl, pH 7.2 |
| buffer B12 | a lysis buffer in RTX-mediated purification | 50 mM Tris-HCl, pH 7.4 |
| buffer B13 | a cleavage buffer in RTX-mediated purification | 20 mM Bis-Tris, 75 mM EGTA, pH 6.2 |
| buffer B14 | a lysis buffer in Annexin B1-mediated purification | phosphate buffered saline (10 mM Na_2_HPO_4_, 2 mM NaH_2_PO_4_, 137 mM NaCl, 2.7 mM KCl, pH 7.4) containing 1 mM phenylmethanesulfonyl fluoride and 1 mg/mL sodium deoxycholate |
| buffer B15 | a cleavage buffer in Annexin B1-mediated purification | 20 mM Bis-Tris, 20 mM EDTA, pH 6.2 |

^a, b^The ionic strengths of buffer B1 and buffer B8 were calculated using the Buffer calculator (https://www.liverpool.ac.uk/pfg/Tools/BuffferCalc/Buffer.html), without considering the trace amount of EDTA in the calculation.

**Reference**

1. Mahmoudi Gomari M, Saraygord-Afshari N, Farsimadan M, Rostami N, Aghamiri S, Farajollahi MM. Opportunities and challenges of the tag-assisted protein purification techniques: Applications in the pharmaceutical industry. Biotechnol. Adv. 2020, 45:107653.

2. Vassylyeva MN, Klyuyev S, Vassylyev AD, Wesson H, Zhang Z, Renfrow MB, Wang H, Higgins NP, Chow LT, Vassylyev DG. Efficient, ultra-high-affinity chromatography in a one-step purification of complex proteins. Proc. Natl. Acad. Sci. U. S. A. 2017, 114:E5138-E5147.

3. Kimple ME, Brill AL, Pasker RL: Overview of affinity tags for protein purification. Curr. Protoc. Protein Sci. 2013, 73:9.9.1-9.9.23.

4. Singh PK, Chan PF, Hibbs MJ, Vazquez MJ, Segura DC, Thomas DA, Theobald AJ, Gallagher KT, Hassan NJ. High-yield production and characterization of biologically active GST-tagged human topoisomerase IIalpha protein in insect cells for the development of a high-throughput assay. Protein Expression Purif. 2011, 76:165-172.

5. Higgins CA, Vermeer LM, Doorn JA, Roman DL. Expression and purification of recombinant human tyrosine hydroxylase as a fusion protein in Escherichia coli. Protein Expression Purif. 2012, 84:219-223.

6. Zheng CF, Simcox T, Xu L, Vaillancourt P. A new expression vector for high level protein production, one step purification and direct isotopic labeling of calmodulin-binding peptide fusion proteins. Gene 1997, 186:55-60.

7. Huyck RW, Keightley A, Laity JH. Expression and purification of full length mouse metal response element binding transcription factor-1 using Pichia pastoris. Protein Expression Purif. 2012, 85:86-93.
